# Strategies for Early Vaccination During Novel Influenza Outbreaks

**DOI:** 10.1038/srep18062

**Published:** 2015-12-14

**Authors:** M. Laskowski, Y. Xiao, N. Charland, S. M. Moghadas

**Affiliations:** 1 Agent-Based Modelling Laboratory, York University, Toronto, Ontario, Canada M3J 1P3; 2 Department of Mathematical Sciences, University of Cincinnati, Cincinnati OH, 45221 USA; 3 Medicago Inc., 1020 Route de l’Église, Bureau 600, Quebec, Quebec, Canada GIV 3V9

## Abstract

Ongoing research and technology developments hold the promise of rapid production and large-scale deployment of strain-specific or cross-protective vaccines for novel influenza viruses. We sought to investigate the impact of early vaccination on age-specific attack rates and evaluate the outcomes of different vaccination strategies that are influenced by the level of single or two-dose vaccine-induced protections. We developed and parameterized an agent-based model for two population demographics of urban and remote areas in Canada. Our results demonstrate that there is a time period before and after the onset of epidemic, during which the outcomes of vaccination strategies may differ significantly and are highly influenced by demographic characteristics. For the urban population, attack rates were lowest for children younger than 5 years of age in all vaccination strategies. However, for the remote population, the lowest attack rates were obtained for adults older than 50 years of age in most strategies. We found that the reduction of attack rates following the start of vaccination campaigns during the epidemic depends critically on the disease transmissibility, suggesting that for a sufficiently high transmissibility, vaccine delivery after the onset of epidemic has little or no effect, regardless of the population demographics.

Vaccination remains the most effective public health measure to prevent influenza infection and its related complications^1^,^2^,^3^. In the absence of this preventive measure, options to mitigate the effects of an influenza pandemic include antiviral therapy and non-pharmaceutical interventions (e.g., social distancing, school closures)^4^,^5^. When vaccine becomes available for a nascent influenza pandemic, countries worldwide will strive to rapidly vaccinate their populations, especially groups identified as ‘high-risk’, to minimize the population burden of the disease^1^,^4^. However, using the conventional egg-based manufacturing method, the large-scale production and distribution of a pandemic vaccine may take up to six months after the identification of the pandemic strain, and thus will not be available during the early stages of disease emergence. In addition, manufacturing capacity is currently insufficient to address the immediate global demand for vaccine supply in the midst of a rapidly spreading disease^2^,^6^.

The 2014–2015 Northern hemisphere influenza season reminds everyone, for yet another time, how efficacy of current vaccines can be affected by a strain mismatch such as the one observed for H3N2. Seasonal influenza vaccine manufacturing using traditional technologies such as egg-based is a time-consuming process^7^. To meet the manufacturing timelines and have vaccines ready for the influenza season in each hemisphere, the World Health Organization (WHO) must make its strain recommendations months in advance by studying the infection patterns in the other hemisphere^7^. This “educated guess” may be challenged by a new strain emerging during the summer, which can diminish the vaccine efficacy. Moreover, a recent study indicated that the low vaccine effectiveness could also be due to the mutations introduced during the egg-based manufacturing process rather than to the antigenic drift in circulating viruses^8^.

To address these global challenges associated with speed and better efficacy, much research and technology development have been devoted to the identification of novel means of vaccine manufacturing that will allow for the rapid production and large-scale deployment of a strain-specific or cross-protective vaccine in the event of an influenza pandemic or emergence of a new strain during yearly epidemics^1^,^3^,^9^,^10^. For example, cell-based vaccines are produced by growing viruses in cultured animal cells^10^. Compared to the conventional egg-based method, this technology relies on cell cultures, which can be cryopreserved and scaled up as required, and fewer mutations occur during the production process^3^. Although cell-based technology produces vaccines with similar safety, immunogenicity, and efficacy compared to egg-based vaccines, it has higher production costs^3^. Subunit vaccines based on the recombinant expression of influenza hemagglutinin (HA) proteins in cultured insect cells are one of the most recent influenza vaccines approved by the US FDA^11^. Although this technology can deliver vaccine doses rapidly, high dosage is required to meet levels of immunological protection^12^, thus limiting the doses available for distribution in case of a pandemic. Deoxyribonucleic acid (DNA)-based technology involves injecting DNA expression vectors related to the nucleotide sequence of the virus into target cells of a host to elicit an immune response^13^. This technology allows for rapid production of influenza vaccines that can be multivalent and induces both antibody- and cell-mediated immunity^3^,^9^. The results of clinical trials show the promise of this technology to provide a higher level of immunological protection compared to egg-based vaccines. However, a major reported drawback is the high amount of DNA required to produce adequate protective levels, which depends on the level of transported DNA into target cells via traditional intramuscular injection^3^,^9^,^13^. Virus-like particles (VLPs) are another technology, which involves viral structural proteins that do not contain any genomic components^3^. Although VLPs cannot replicate, they are structurally similar to viruses and can thereby elicit a more broadly effective immune response in the host^3^. Influenza VLPs have been previously produced from multiple expression systems, including baculovirus, vaccinia virus, and plant-based systems^3^. VLP-based vaccine technology has the potential to rapidly produce safe and cost-effective vaccines, and the efficacy of these vaccines is currently being evaluated in humans^3^,^14^,^15^. In addition to these production methods, a number of other vaccine technologies, such as virus-vectored approaches are in various stages of research and clinical trials, with the potential for rapid and large-scale production of influenza vaccines^3^,^9^.

While each vaccine technology has various benefits and drawbacks, these technologies show significant promise to address the challenges of conventional egg-based vaccine production. This enables the development of a vaccine for testing and distribution possible within a few weeks following the identification and genetic sequencing of a new influenza strain, whether pandemic or emerging shortly before the yearly influenza season^3^,^16^. Early vaccination has several potential benefits from both public health and socioeconomic perspectives, including reduced rates of infection, hospitalization, and death, along with reduced stress on the healthcare system.

Given the availability of a strain-specific or cross-protective vaccine during the early stages of a pandemic or seasonal epidemic, we sought to evaluate the effectiveness of different vaccination strategies in reducing attack rates in the population. For this evaluation, we developed an agent-based simulation model from a previously established framework to include the effect of vaccination. For vaccination strategies evaluated here, we considered the possibility that the first dose of vaccine may provide low protection levels in all age groups, and therefore a second dose would be required during a pandemic. Considering the time for start of vaccination compared to the onset of epidemic (defined as the time for identification of the first clinical case of influenza in a specific population), and the lag-time between the first and second vaccine doses in the case of low-efficacy vaccines, we specifically addressed the following questions:

- Does the level of vaccine-induced protection influence the outcomes of vaccination strategies?
- How significantly do vaccination strategies differ with delay in vaccine availability?
- What is the impact of vaccination strategies on the reduction of hospitalizations?

## Results

All individuals were considered eligible to receive vaccine during the outbreak, except those who were identified as infectious cases. In the model context, infectious cases are identified when they seek and receive care (i.e., treatment or hospitalization) with a probability that varied in simulations. For the implementation of vaccination, we included two key parameters: the time at which the vaccination campaign starts and the efficacy of vaccine. The time for start of the vaccination campaign was in the window of 2 weeks before the identification of the first infectious case to 4 weeks after the onset of epidemic in the population. Since vaccination may not be 100% effective, especially against a pandemic strain^17^,^18^,^19^,^20^,^21^, we considered two scenarios: a single-dose vaccination where vaccine is highly effective, and a two-dose vaccination where the first dose confers a low protection. For each vaccinated individual, the protection level was randomly selected in the estimated range, which is age-dependent (Table S1). For the scenario of a two-dose vaccination, we considered a three-week delay between the first and second doses. We simulated two population settings with demographics corresponding to an urban centre and a remote community in Canada. The baseline reproduction numbers were 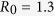
22,23,24 for urban and 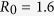
22,23 for remote populations. Results for higher reproduction numbers are provided in Supplementary Materials.

### Single-dose vaccination strategies in urban population

**Figure 1:**
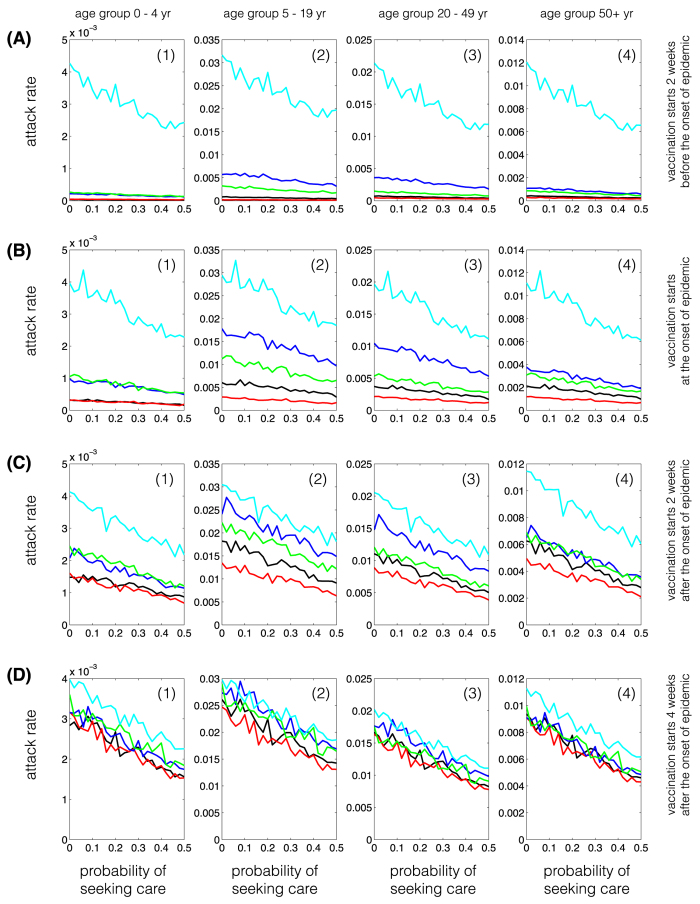
Age-specific attack rates for single-dose vaccination strategies in urban centre. Curves correspond to the scenarios without vaccination (cyan), and the morbidity-based (red), risk-based (black), outcome-based (blue), and random (green) vaccination strategies. The horizontal axis represents the fraction of symptomatically infected individuals who seek care during symptomatic infection. The vertical axis represents the fraction of population infected (symptomatically or asymptomatically) throughout the epidemic.

When the vaccination campaign starts two weeks before the onset of epidemic (Fig. 1, A(1)–A(4)), attack rates are lowest in all age groups for the morbidity-based strategy (red curves), which prioritizes school-aged children as the first group for vaccination. The next best strategy in reducing attack rates is the risk-based strategy (black curves). The outcome-based (blue curves) and random vaccination strategies (green curves) lead to comparable attack rates in the 0–4 and 50+ year age groups. However, random vaccination outperforms the outcome-based strategy for the 5–19 and 20–49 years age groups.

**Figure 2:**
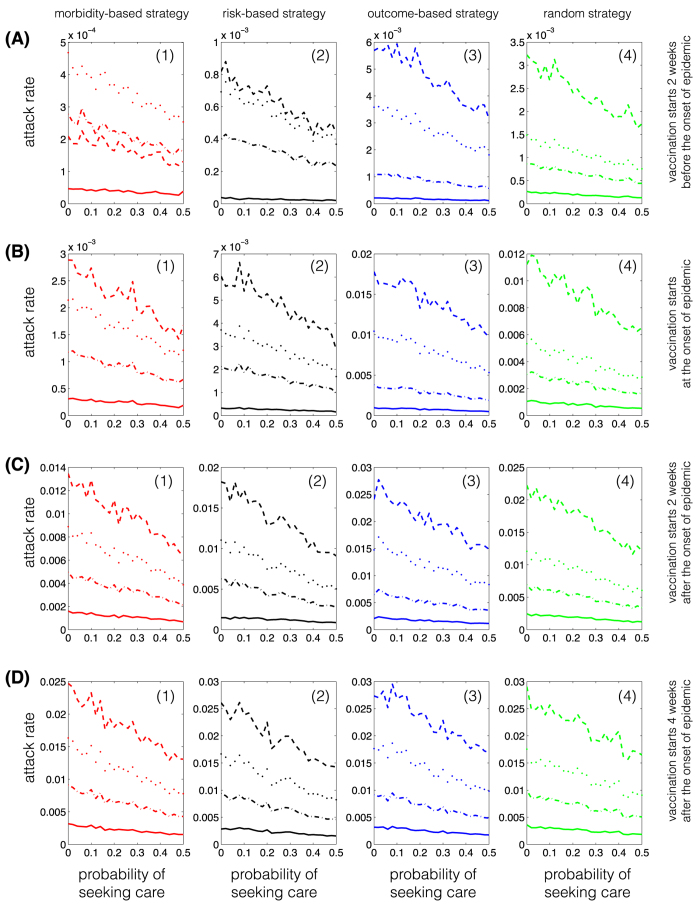
Attack rates for single-dose vaccination strategies in urban centre. Curves correspond to age groups 0–4 (solid); 5–19 (dashed); 20–49 (dotted); and 50+ (dot-dashed). Colours correspond to the morbidity-based (red), risk-based (black), outcome-based (blue), and random (green) vaccination strategies. The horizontal axis represents the fraction of individuals who are symptomatically infected and seek care during symptomatic infection. The vertical axis represents the fraction of population in different age groups infected (symptomatically or asymptomatically) throughout the epidemic.

We observed similar outcomes when the vaccination campaign starts at the beginning of epidemic, or two and four weeks after the onset of epidemic (Fig. 1, B(1)–B(4), C(1)–C(4), D(1)–(4)). However, the effect of vaccination on lowering attack rates diminishes with delay in the start of the vaccination campaign. For a delay more than four weeks in the start of vaccination, the choice of strategy has virtually no impact on the age-specific attack rates. In all scenarios simulated here, the morbidity-based strategy remains the most effective strategy in all age groups (Fig. 2). Similar outcomes were achieved for the relative age-specific attack rates for different vaccination strategies (Figs S6 and S7).

For the morbidity-based strategy, attack rates in the 20–49 years age group are higher than other age groups when the vaccination campaign starts two weeks before the onset of epidemic (Fig. 2 A(1)). However, when it starts during the epidemic, attack rates are highest among 5–19 years age group. For other vaccine strategies, attack rates from highest to lowest belong to the 5–15, 20–49, 50+, and 0–4 years age groups respectively, regardless of the time at which the vaccination campaign begins (Fig. 2).

### Two-dose vaccination strategies in urban population

When the vaccination campaign starts 2 weeks before the onset of epidemic, we observed similar outcomes to those obtained for a vaccine with high efficacy and single dose for different strategies (Figs S8–S11). We found that attack rates in the 0–4 years age group were comparable between the morbidity-based (red curves) and risk-based strategies (black curves) (Fig. S8). Attack rates of the outcome-based strategy (blue curves) were higher or equivalent to the random strategy (green curves) in all age groups, regardless of the time for start of vaccination. When the vaccination campaign starts four weeks after the onset of the epidemic, we did not observe any significant difference between the projected attack rates in different vaccination strategies, with little or no effect in reducing attack rates compared to the scenario without vaccination (Figs S8 and S9).

### Single-dose vaccination strategies with shifted demographics

**Figure 3:**
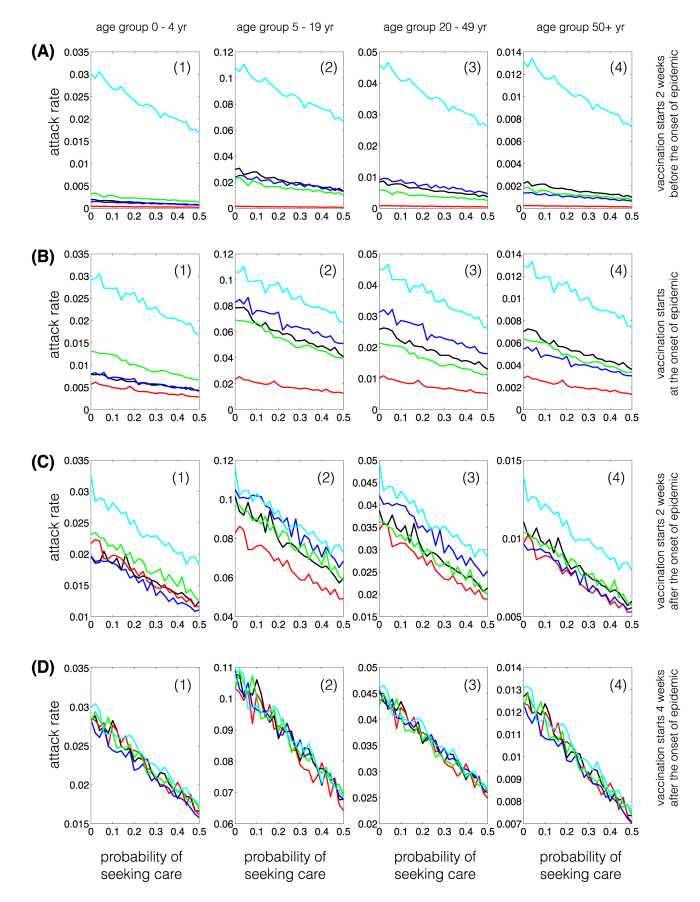
Age-specific attack rates for single-dose vaccination strategies in shifted demographics (remote community). Curves correspond to the scenarios without vaccination (cyan), and the morbidity-based (red), risk-based (black), outcome-based (blue), and random (green) vaccination strategies. The horizontal axis represents the fraction of symptomatically infected individuals who seek care during symptomatic infection. The vertical axis represents the fraction of population infected (symptomatically or asymptomatically) throughout the epidemic.

**Figure 4:**
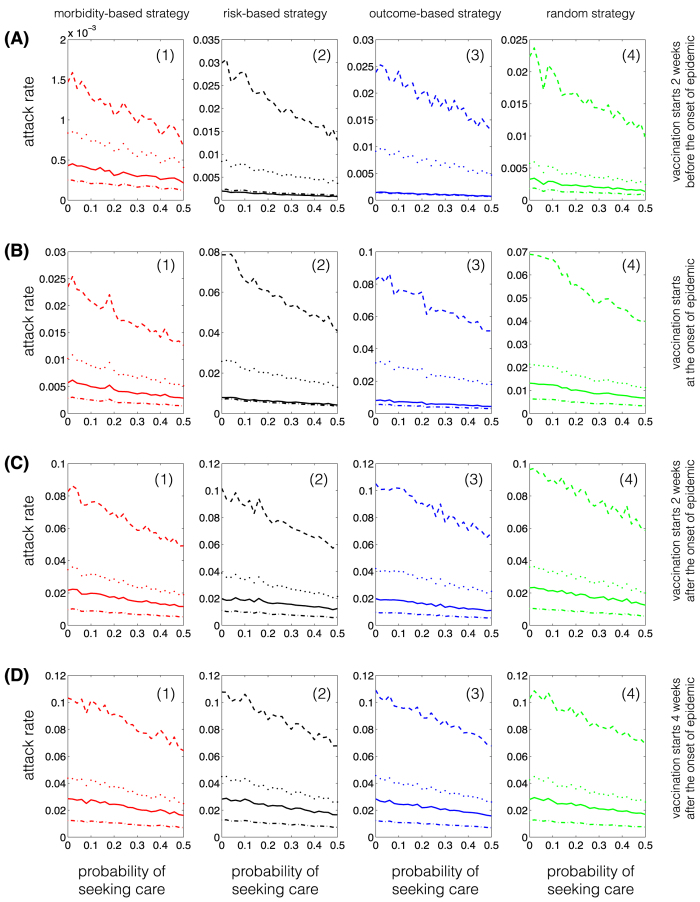
Attack rates for single-dose vaccination strategies in shifted demographics (remote community). Curves correspond to age groups 0–4 (solid); 5–19 (dashed); 20–49 (dotted); and 50+ (dot-dashed). Colours correspond to the morbidity-based (red), risk-based (black), outcome-based (blue), and random (green) vaccination strategies. The horizontal axis represents the fraction of symptomatically infected individuals who seek care during symptomatic infection. The vertical axis represents the fraction of population in different age groups infected (symptomatically or asymptomatically) throughout the epidemic.

Compared to the urban demographics, we observed a number of differences between vaccination strategies for reduction of attack rates in different age groups. When the vaccination campaign starts two weeks before the onset of epidemic (Fig. 3 A(1)–A(4)), age-specific attack rates are lowest in all age groups for the morbidity-based strategy, and comparable for other strategies. In contrast, when the vaccination campaign starts at the beginning or after the onset of epidemic, the morbidity-based strategy does not necessarily lead to the lowest attack rates in all age groups. In the 0–4 years age group, the attack rate in the risk-based strategy is comparable to, or slightly lower than, that obtained using the morbidity-based strategy (Fig. 3 C(1)). For a later start of vaccination campaign, we do not observe any significant differences between various strategies; however, the outcome-based strategy appears to have a slight advantage in reducing attack rates in the youngest and oldest age groups (Fig. 3C(1),C(4),D(1),D(4)), which contrasts the results of the corresponding scenarios for the urban demographics (Fig. 1C,D). Furthermore, attack rates from highest to lowest belong to the 5–19, 20–49, 0–4, and 50+ years age groups respectively in the morbidity-based and random vaccination strategies regardless of the start of vaccination (Fig. 4). However, for the risk-based and outcome-based strategies, attack rates among the 0–4 years age group is lower than, or equivalent to, those for the 50+ years group age when vaccination starts before the onset of epidemic. These observations hold for the relative attack rates in the shifted demographics for different vaccination strategies (Figs S12 and S13).

### Two-dose vaccination strategies shifted demographics

For two-dose vaccination strategies, we obtained attack rates that have qualitatively similar patterns to those obtained in single-dose vaccination strategies. The outcomes of these strategies for different age groups with respect to the time for start of the vaccination campaign are illustrated in Figs S14–S17.

### Reduction of hospitalization

**Figure 5:**
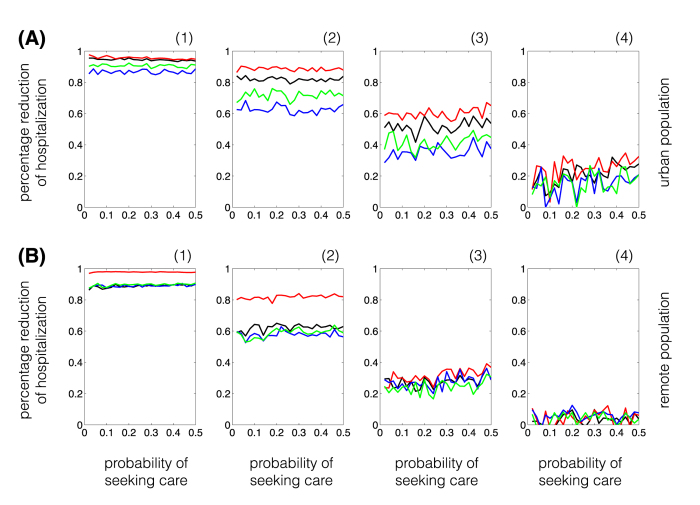
Percentage reduction of hospitalization for different vaccination strategies compared to the scenario without vaccination. Panel (**A**) and (**B**) correspond to single-dose vaccination strategies in urban 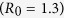 and remote 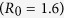 populations, respectively, with the start vaccination (1): two weeks before the onset of epidemic; (2): at the onset of epidemic; (3) two weeks after the onset of epidemic; and (4): four weeks after the onset of epidemic. Colours correspond to the morbidity-based (red), risk-based (black), outcome-based (blue), and random (green) vaccination strategies. The horizontal axis represents the fraction of symptomatically infected individuals who seek care during symptomatic infection. On vertical axis, the range 0–1 corresponds to 0–100% reduction of hospitalization.

We obtained the percentage reduction of hospitalization by comparing each vaccination strategy to the baseline scenario without vaccination. Figure 5 shows that, for single-dose vaccination, the morbidity-based strategy leads to the highest reduction of hospitalization in both urban and remote populations. Surprisingly, while the outcome-based strategy has a higher reduction of hospitalization in the 0–4 and 50+ years age groups (Fig. S18), it has the lowest reduction in the overall hospitalizations compared to other vaccination strategies (Fig. 5). We found that, depending on the time for the start of the vaccination campaign, the risk-based strategy results in higher or comparable reduction of hospitalization than random strategy in the urban population; however, this outcome is reversed in the remote population. Similar outcomes were qualitatively observed for two-dose vaccination strategies using low-efficacy vaccines, with a significantly lower percentage reduction of hospitalization (Figs S19 and S20).

## Discussion

Our study for the first time provides a comparative evaluation of vaccination strategies with the time of implementation (with respect to the onset of disease outbreak in the population) for mitigating the impact of a novel influenza virus. Our comparisons across population age groups have several important implications for vaccination policies. First and foremost is the fact that for a sufficiently high reproduction number, starting the vaccination campaign after the onset of epidemic has little or no effect in reducing attack rates, regardless of the population demographics. Even for relatively low reproduction numbers comparable to seasonal influenza, if the vaccination campaign begins too late with respect to the onset of the epidemic, then attack rates remain independent of the choice of vaccination strategy. Estimates of the reproduction number of a pandemic strain may therefore be essential for determining the most effective vaccination policies. Second, early vaccination, and optimally before the onset of disease outbreak, leads to lowest attack rates for both high and low efficacy vaccines, which highlights the importance of timely availability of vaccine. We observed that for early vaccination, a strategy that aims to reduce transmissibility of the disease in the population (i.e., morbidity-based) has the highest impact on lowering attack rates in all age groups, and provides the highest percentage reduction of hospitalization compared to other vaccination strategies. However, there is a time period before and after the onset of epidemic, during which the outcomes of vaccination strategies may differ significantly. Furthermore, demographic variables could influence these outcomes, suggesting that vaccination policies should be tailored to fit the demographic and geographic characteristics of the populations.

In the event of an influenza pandemic, timelines for the global spread of the disease may still be too short to allow for large-scale vaccine production, distribution, and implementation during the first pandemic wave. However, technologies that can deliver the first doses of vaccines within 12 weeks after the identification of a new pandemic strain could play a significant role for mitigating disease outcomes in subsequent pandemic waves^25^. In this study, we considered a time-lag of three weeks between the first and second vaccine doses in strategies evaluated for low-efficacy vaccines. Previous studies have considered a longer time-lag (four weeks)^26^, which would lead to even lower impact on reducing attack rates. However, a recent randomized trial of H5N1 adjuvanted vaccine suggests that accelerated immunization schedules with a short time-lag (7 or 14 days) between the first and second doses may be effective in enhancing vaccine efficacy and help gain early control of influenza pandemics^27^. Our simulations corroborate these effects in the population, demonstrating that as the time-lag between vaccine doses reduces, the impact of vaccination becomes more pronounced on reducing attack rates. For a short delay in administration of the second dose of vaccine, the outcomes of two-dose strategies with low-efficacy vaccines approach those of single-dose vaccination with high-efficacy vaccines.

Our findings demonstrate that the choice of vaccination strategy is highly important when vaccines become available during the critical period around the onset of the epidemic, a volatile time with much uncertainty about the disease and its potential impact on the population. To date, no study has assessed the determinants (e.g., acceptability and potential uptake rates) of vaccination produced using a novel technology with early distribution during an outbreak, although such technologies will be the reality for vaccine production in the near future. This assessment, while beyond the scope of this study, is essential to inform vaccination policies and optimize roll-out strategies that could maximize the population-wide benefits of early vaccination by raising herd immunity.

Vaccination scheduling is also important for vaccines against annual epidemics as a strain mismatch can have significant impact on vaccine efficacy. The use of newer technologies to manufacture influenza vaccines in shorter timelines compared to the traditional egg-based method could allow the WHO to delay strain recommendations to be included in a vaccine, and yet still have the vaccines ready ahead of the influenza season to allow for optimal planning of vaccination campaigns.

Our model combines the available information, data, and evidence in order to investigate the synergistic effects of vaccine, treatment, and isolation strategies. However, there are limitations that should be considered for interpretation of the results. The structure of the model for contact patterns is based on reasonable, general assumptions about human behavior consistent with those used in previous work^28^, but no specific databases are available for detailed contact network data and behavioural patterns for population settings simulated here. Specifically, workplace contact patterns in industrialized countries are poorly understood across occupations. We assumed a uniform susceptibility across all age groups in the population that is completely naïve to the emerging influenza virus. However, for emerging descendant viruses such as H1N1pdm09, different age groups in the population may render differential susceptibility as a result of pre-existing immunity^29^,^30^. We also assumed that there is no limitation on the vaccine quantities, and vaccine demand exceeds the availability of vaccine, resulting in a high uptake rate. However, uptake rates may be affected by several factors in addition to the availability of vaccines, such as timelines for prioritization and individuals’ perception of the risk of infection, as was observed during the 2009 pandemic^31^. Simulation results reported here are based on a daily distribution rate of vaccines given in Table S1. We also investigated scenarios with increased and decreased rates of vaccine distribution, and observed that the results remain qualitatively similar for different vaccination strategies. However, as expected, the magnitude of attack rates is reduced (increased) for higher (lower) vaccine distribution rates. We parameterized the model for disease outcomes, treatment, and hospitalization based on laboratory confirmed case counts collected for the 2009 pandemic in the simulated populations (Fig. S37). However, the associated rates could vary between populations, and may depend on demographic characteristics, public health capacity, access to healthcare, and other health conditions^32^. In the absence of detailed data for the age-specific probability of infected individuals seeking care (which may be affected by a number of factors including the severity of the disease), we assumed the same probability of seeking treatment across all age groups. We also parameterized the model with estimates of vaccine effectiveness reported for seasonal influenza vaccination^17^,^18^,^19^,^20^,^21^, and used previous estimates for the effect of antiviral drugs as a reduction of disease transmissibility following the start of treatment^33^,^34^,^35^. We assumed age-specific probabilities of self-isolation derived from previous literature to parameterize the model. However, the degree to which individuals practice self-isolation and the effect of self-isolation in the reduction of disease transmissibility depends on a number of factors, including contact patterns, behavioural responses, and severity of the disease.

For simulated vaccination strategies, we relied on previous reports and data pertaining to the populations studied here. For example, for the morbidity-based strategy, it is assumed that most transmission occurs through contacts with young (5–19 and 20–49 years) age groups, which were prioritized in the model simulations. However, in the context of vaccination, the degree to which disrupting disease transmission through vaccination of younger individuals would affect morbidity and disease outcomes in those at high risk of infection remains undetermined. The risk-based strategy has some realistic limitations on the availability of information when deciding on which age groups to prioritize for vaccination. We assumed that the counts of confirmed cases in each age group are delayed by up to two weeks, and considered only individuals who seek care. While frequent changes in vaccine prioritization can be implemented during an outbreak^31^, its practical challenges are numerous.

Despite these limitations, our results remain robust in projecting that there is no significant difference in attack rates obtained from different vaccination strategies when vaccination starts sufficiently late after the onset of epidemic. For early vaccination, however, the choice of vaccination strategy can have a significant impact on attack rates in the population, underscoring the importance of research and development in technologies for rapid vaccine production against novel influenza viruses.

## Materials and Methods

### Modelling structure

A previously validated agent-based model^28^,^35^ was used as the basic framework for the spatiotemporal interactions between individuals. We extended this framework to include vaccination and project age-specific attack rates using different vaccination strategies. The model was initialized with individual agent characteristics that are drawn from pertinent demographic distributions for age, gender, and household compositions. The environment includes residences for agents (homes), workplaces, schools and public places, where agents can be located according to the schedule module of the simulations. Agent schedules define relationships between agents and positions in the lattice environment as a function of time, permitting a probabilistic spread of disease between co-located agents. The schedules dictate agent relationships (that are changing in time) with particular positions in the lattice, and include random movements that resemble a Levy-flight^36^, with the probability of being present at a more distant position on the next time step of the simulations decreasing exponentially. Initially, all agents were assumed to be susceptible with no pre-exiting immunity. In each simulation scenario, an infectious agent was randomly chosen as the initial infection, and agent disease states were updated in increments of 1 hour as the unit of time in simulations. All simulations were carried out on the Compute Canada mp2 compute cluster located at Université de Sherbrooke. Each set of 1000 realizations required approximately 16 hours of a single CPU core utilizing about 20 MB of memory, on average.

### Population study

We implemented the model for an urban centre in the province of Manitoba, Canada. To evaluate the effect of demographic and geographic variables, we also simulated the model with shifted demographics to resemble a remote community in northern Manitoba. Statistics Canada census data^37^,^38^, rounded to the nearest five individuals, were used as a basis for the number of households, individuals per household, and individuals’ ages, genders, and employment characteristics (Figs S3–S5). In the model simulations, age is considered as a continuous variable. However, in order to convey the results amenable to the public health context, we defined four age groups as: 0–5 (pre-school); 5–19 (school children); 20–49 (young adults); and 50+ (older adults).

### Disease natural history

The model consists of different states corresponding to a conceptual framework developed for the natural history of influenza infection^39^, as well as interventions including treatment, hospitalization, vaccination, and self-isolation. Disease natural history states include stages of latent (not yet infectious), pre-symptomatic (infectious before clinical manifestations), symptomatic (infectious with clinical symptoms), and asymptomatic (infectious without showing clinical symptoms)^39^. In the simulation model, if transmission occurs, an infected individual will first complete an incubation period, which combines the latent and pre-symptomatic stages. The newly infected individual either continues the course of infection by developing symptomatic infection, or exhibits asymptomatic infection. Interventions of treatment and hospitalization in the model were offered only to those individuals who sought care during symptomatic infection, and identified to be treated or hospitalized. Only individuals with symptomatic infection may practice self-isolation. Epidemiological states of the individual agents in the model are summarized in Table S2.

### Disease transmission

Considering the interaction of two individuals, one susceptible and the other infectious, disease transmission occurs as a result of rejection sampling based trials where the chance of success is defined by a transmission probability distribution^28^. This probability is a disease specific parameter, which may be influenced by interventions. For example, a vaccinated individual may be subject to a lower transmission probability (depending on the level of immunity) compared to a fully susceptible individual.

The probability of disease transmission between each pair of infectious and susceptible individuals co-located in the environment for the duration of 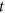 time-steps was calculated by


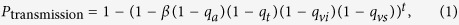


where *β* is the baseline transmission rate per time-step, 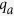 is the reduced transmissibility of an infectious individual in the asymptomatic or pre-symptomatic state compared to symptomatic state, 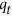 is the reduction in transmissibility following the start of an effective course of antiviral treatment during symptomatic infection, 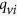 is the vaccine-induced protection level in the infectious individuals, and 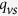 is the vaccine-induced protection level in the susceptible individual at the time of exposure. The parameter *β* was obtained by calibrating the model to achieve an estimated reproduction number 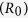 by observing the average number of secondary cases generated by the initial infectious case, over 1000 independent realizations. This calibration was carried out in the absence of control measures.

### Treatment and hospitalization

Individuals with symptomatic infection may seek care with the probability 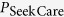, a model parameter that was varied between 0 and 0.5. Individuals who seek care may require hospitalization, and the probability of hospitalization was age-dependent obtained from epidemiological (laboratory confirmed cases) data collected during H1N1pdm09 in the province of Manitoba, Canada (Fig. S37). Hospitalized individuals are considered to be in isolation until recovered, and therefore cannot infect other individuals. We considered antiviral treatment for those who sought care, but are not hospitalized, and treatment continued until they recover. In this model, individuals who do not seek care are classified as unidentified infections.

### Vaccination

Individuals who received vaccines (in either single-dose or two-dose strategy) may become infected, but at a reduced rate of acquiring infection compared to unvaccinated individuals. The probability of transmission depends on the vaccine-induced protection level at the time of exposure. We assumed a linear increase in the vaccine-induced protection to the sampled level over a two-week period following vaccination (Fig. S1).

We assumed that a certain number of vaccine doses are available per day (Table S1), and the supply of vaccine remains constant throughout the outbreak. Following the commencement of the vaccination campaign, for each hour that vaccine is being distributed, a number of individuals from the population receive the vaccine, according to the vaccine prioritization strategy. We assumed that vaccinated individuals who become infected are less likely to develop clinical symptoms (with an increased probability of being asymptomatic) due to the immunological effects of vaccination. Furthermore, for a two-dose strategy, a second dose was given only to those who received the first dose. We considered possibility of drop-out from the vaccination program, either due to acquiring infection after receiving the first dose, or simply due to individuals voluntarily forgoing the second dose.

Four different vaccine prioritization strategies were considered:

- *Random*: the probability of vaccination in a particular age group depends only on the number of eligible, unvaccinated individuals in that age group.
- *Morbidity-based*: prioritization of age groups is based on the highest attack rates observed in previous studies. The first priority group was school-aged children (i.e., the 5–19 years of age group).
- *Outcome-based*: prioritization of age groups is based on the highest rates of hospitalization observed in laboratory confirmed data collected during H1N1pdm09 in the province of Manitoba, Canada. The first priority groups were pre-school children (0–4 years of age) and older adults (50+ years of age).
- *Risk-based*: prioritization of age groups is probabilistically based on estimates of the risk within each age group. This risk is calculated using cumulative confirmed case counts within the model for each week during the pandemic:

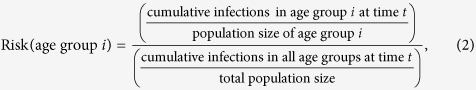


Vaccination of age groups is then prioritized proportional to their risk.

In all strategies, vaccination of the next priority group was initiated when there were no more eligible individuals in the current priority group. For vaccination campaigns beginning before there are any confirmed cases, prioritization is implemented the same as in the morbidity-based strategy. The priority order of age groups for vaccination is detailed in Supplementary Materials.

### Parameterization

**Figure 6:**
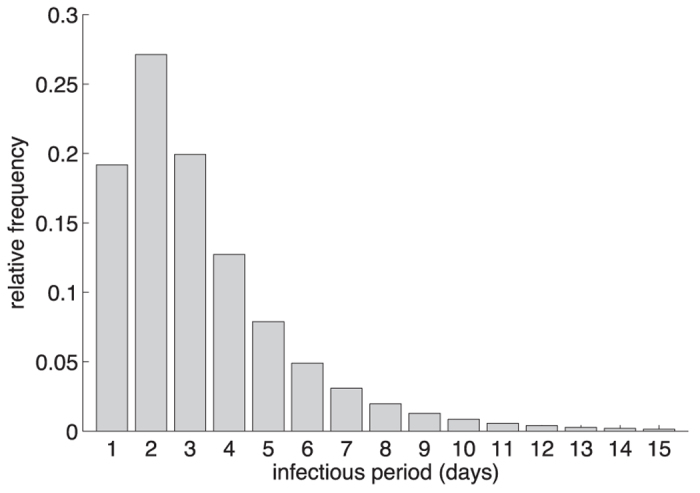
Log-normal distribution for the relative frequency of infectious periods.

We parameterized simulations with transmission rates that were calibrated to estimates of the reproduction number^22^,^23^,^24^. Parameters associated with the natural history of disease were sampled from relevant distributions for each individual independently. The latent period was drawn from a uniform distribution with a minimum of 1 day and a maximum of 2 days^33^,^40^. The pre-symptomatic period for each infected individual was drawn from a log-normal distribution with the scale parameter *μ* = −0.775 days, and shape parameter 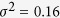 days, giving an average of 0.5 days^28^,^41^. The duration of infection following the start of clinical symptoms was sampled from a log-normal distribution (Fig. 6), with the scale parameter *μ* = 1 day, and the shape parameter 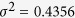 days, which has a mean of 3.38 days^22^,^23^,^24^. Asymptomatic infection was assumed to be 50% less infectious than symptomatic infection^33^,^42^. We ran simulations for the range 0–0.5 of 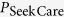 for individuals seeking care after developing clinical symptoms. The delay in seeking care was randomly sampled for each symptomatic individual independently from the uniform distribution in the range 0.5–3.5 days^32^ after the onset of symptoms. The age-dependent probabilities of hospitalization and treatment were calculated from laboratory confirmed data of H1N1pdm09 collected for the health region containing the population study in Manitoba, Canada (Fig. S37).

The effect of antiviral treatment in reducing infectiousness was included as a reduction factor for disease transmissibility following the initiation of treatment for symptomatic infections. We assumed that treatment reduces the transmissibility by 60%^33^,^34^,^35^,^41^,^42^. The protection level of vaccination for each individual was sampled from the estimated ranges for vaccine effectiveness associated with each age group. The mean protection level induced by a single dose of a high efficacy vaccine was 80%. For a vaccine with low efficacy, the mean protection level of the first dose was 30%, and increased to a mean level of 80% for the second dose^13^. The associated ranges for different age groups are provided in Table S1.

### Simulations and comparisons

To evaluate vaccination strategies, we compared age-specific attack rates in simulated scenarios. Simulations were seeded with a randomly selected initial infection when vaccination starts 2 weeks before the onset of epidemic, at the onset of epidemic, and 2 and 4 weeks after the start of epidemic. For the urban centre, we assumed the reproduction numbers 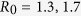 within the estimated ranges for the 2009 H1N1 pandemic^22^,^23^,^24^. We used higher reproduction numbers 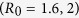 for simulating scenarios with shifted demographics to resemble a remote community^22^,^23^. To capture the effect of changes in transmissibility of the disease and time for the start of vaccination, we calibrated and simulated the model for various reproduction numbers (see Supplementary Materials; Figs S21–S36). For a high efficacy vaccine, only a single-dose of vaccine was implemented in different strategies, with the probability of seeking care in the range 0–0.5. For a vaccine with low efficacy, we implemented a two-dose strategy (Figs S8–S11, S14–S17), assuming that 80% of the individuals vaccinated with the first dose will receive the second dose of vaccine three weeks after the first dose^43^,^44^,^45^. Simulation outcomes were analyzed for age-specific attack rates (the fraction of the total number of infections in each age group to the total number of infections in the population throughout the epidemic) and age-specific relative attack rates (the fraction of the total number of infections in an age group to the total population size of the same age group throughout the epidemic). For comparison purposes, we analyzed outcomes in two graphical representations: (i) the effect of different strategies on each age group, and (ii) the effect of each strategy on different age groups.

## Additional Information

**How to cite this article**: Laskowski, M. *et al.* Strategies for Early Vaccination During Novel Influenza Outbreaks. *Sci. Rep.* **5**, 18062; doi: 10.1038/srep18062 (2015).

## Supplementary Material

Supplementary Information
