# Supplementary material for: Strategies for Early Vaccination During Novel Influenza Outbreaks
Source: Sci Rep. 2015 Dec 14;5:18062. doi: 10.1038/srep18062 (PMC4677284; doi:10.1038/srep18062)
Supplement: Supplementary Information [file srep18062-s1.pdf]

Supplementary Materials
Strategies for Early Vaccination During Novel Influenza Outbreaks

Marek Laskowski,[1] Yanyu Xiao,[2] Nathalie Charland,[3] Seyed M. Moghadas[1]
[1] Agent-Based Modelling Laboratory, York University, Toronto, Ontario M3J 1P3, Canada.
[2] Department of Mathematical Sciences, University of Cincinnati, Cincinnati OH, 45221 USA
[3] Medicago Inc., 1020 Route de l'Église, Bureau 600, Quebec, Quebec, Canada, GIV 3V9.

This supplementary information provides details of the model structure, its implementation, and parameter values (ranges) used for simulations. It also provides figures supporting the results presented in the main text for $R_0 = 1.3$ (22-24) and $R_0 = 1.6$ (22,23) in urban and remote populations, respectively. Results for higher R_0 were found to have similar qualitative behavior and are provided for $R_0 = 1.7$ and $R_0 = 2$ in urban and remote populations, respectively.

1. Details of model implementation

This model was based on the general framework for disease spread (28) with the inclusion of treatment for symptomatic infection (41). We included the effect of vaccine in the model as a reduced probability of acquiring infection for vaccinated individuals, as well as reduced probability of developing clinical symptoms if infection occurs.

For model calibration we averaged over 1000 realizations at various transmission rates (probability of transmission per unit time) counting the number of secondary symptomatic cases to determine R_0 in the absence of any intervention. For each realization a different random individual was selected as the initial case.

Data points for results in all figures were the average of 1000 realizations in which there was at least one secondary case. Realizations in which there were no secondary infections were discarded. Each realization was run with a random initial infection, which was allowed to be a vaccinated individual if vaccination started before the onset of epidemic.

The protection efficacy of vaccine was randomly sampled for each individual from the estimated ranges summarized in Table S1. In case of low vaccine efficacy, where a two-dose strategy was implemented, we considered a three-week time period between the administration of the first and second doses of vaccine. The protection efficacy of vaccine was assumed to linearly increase to the sampled level for each individual following first and second doses, as illustrated in Figure S1.

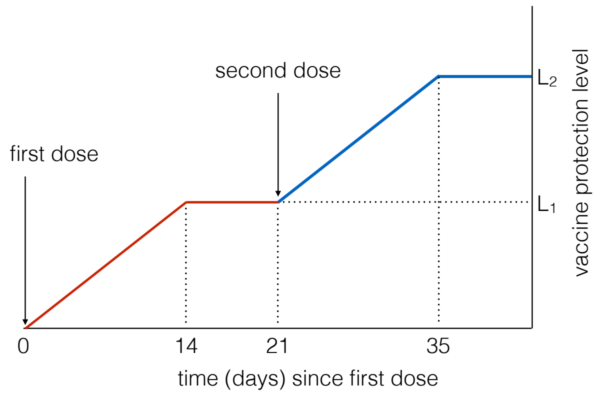

Figure S1. Protection levels resulting from first and second doses of vaccine. Protection level rises linearly until it reaches the sampled level L_1 after 14 days. In scenarios with high efficacy vaccine, a second dose was not required, and thus the individual's protection level remains at L_1 after vaccination. For low efficacy vaccine, a second dose may be given 21 days after the first dose. In this case, the protection level increases linearly from L_1 to the second sampled level L_2 over 14 days, and remains at L_2 (27).

2. Vaccination strategy prioritization

The priority order of age groups for vaccination is:

- Morbidity-based strategy: first 5 – 19, then 20 – 49, and lastly 0 – 4 and 50+.
- Outcome-based strategy: first 0 – 4 and 50+, then 5 – 19, and lastly 20 – 49.

For prioritization in the risk-based strategy, we used the probability

$$P_i = \frac{\left(\dfrac{\text{Risk for age group } i}{\sum_{j=1}^{4} \text{Risk for age group } j} \right)}{\text{number of unvaccinated, untreated individuals in age group } i}, \quad \text{(A1)}$$

for vaccination of individuals in age group i, where the 'Risk' for an age group is defined in the main text.

3. Disease natural history

The model for the natural history of the disease has been previously described in Figure 1 of (39) considering Susceptible, Exposed, Pre-symptomatic, Infectious (both Symptomatic and Asymptomatic), and Recovered states. The probability of developing symptoms is

$$P_{\text{symptomatic}} = (1 - P_A)(1 - q_{vi}), \quad \text{(A2)}$$

where P_A is the probability of developing asymptomatic infection in the absence of vaccine (see Table S1), and q_{vi} is the current vaccine protection level in the individual.

4. Treatment and hospitalization model

Symptomatic infections may receive care (considered as seeking care) in the form of treatment only, or hospitalization (Figure S2). Individuals that are not hospitalized (whether sought care or not) may practice self-isolation. Hospitalized individuals are completely isolated.

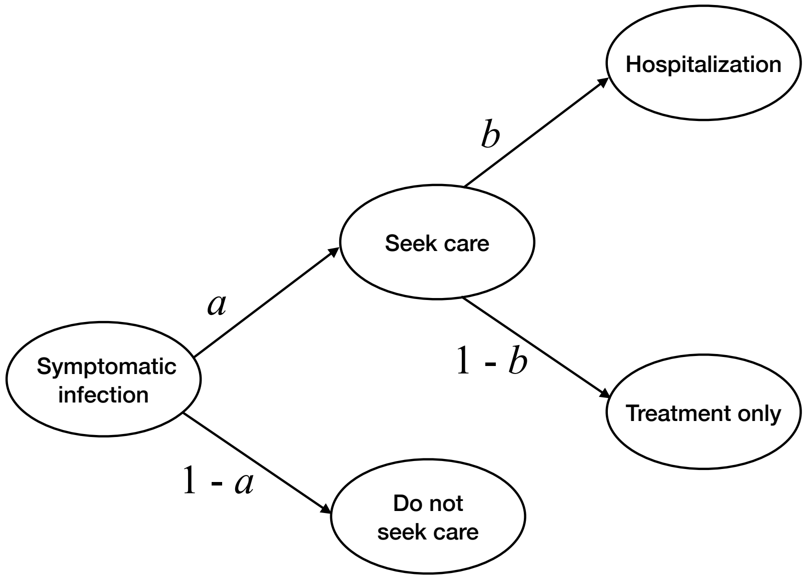

Figure S2. Hospitalization and treatment model. Symptomatic individuals seek care with probability *a* (described in Table S1). Individuals who seek care are hospitalized with probability *b* (described in Table S1). Individuals who seek care but are not hospitalized receive antiviral treatment only.

5. Demographic distributions

We used census databases of Statistics Canada to derive distributions of age, household compositions, and employment rates in both urban centre and shifted demographics (remote community). The rates for urban centre were scaled down by a factor of 366 (that is the ratio of the total population sizes of urban to remote). These distributions are given in figures below.

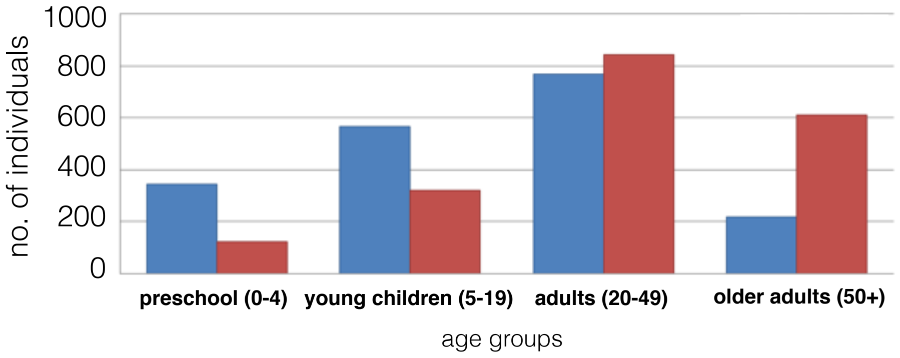

Figure S3. Comparison of age group sizes between urban (red bars; scaled population size) and remote (blue bars) populations.

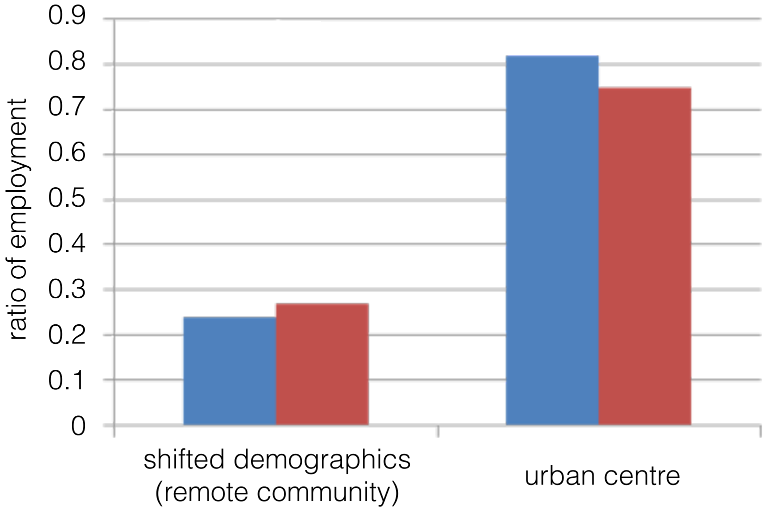

Figure S4. Comparison of ratio of employed males (blue bars) and females (red bars) to all working-age in both urban and remote populations.

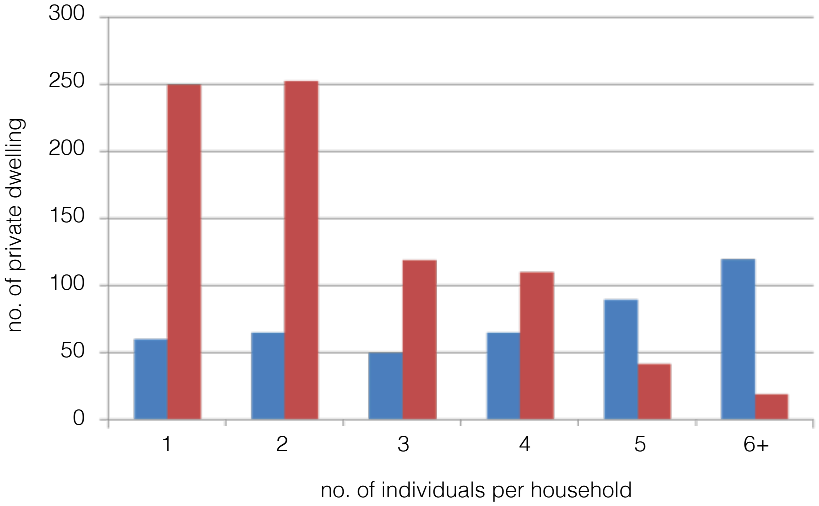

Figure S5. Comparison of household sizes between urban (red bars) and remote (blue bars) populations.

6. Simulations for urban centre with $R_0 = 1.3$

Simulations presented in this section correspond to the results presented in the main text for the urban population.

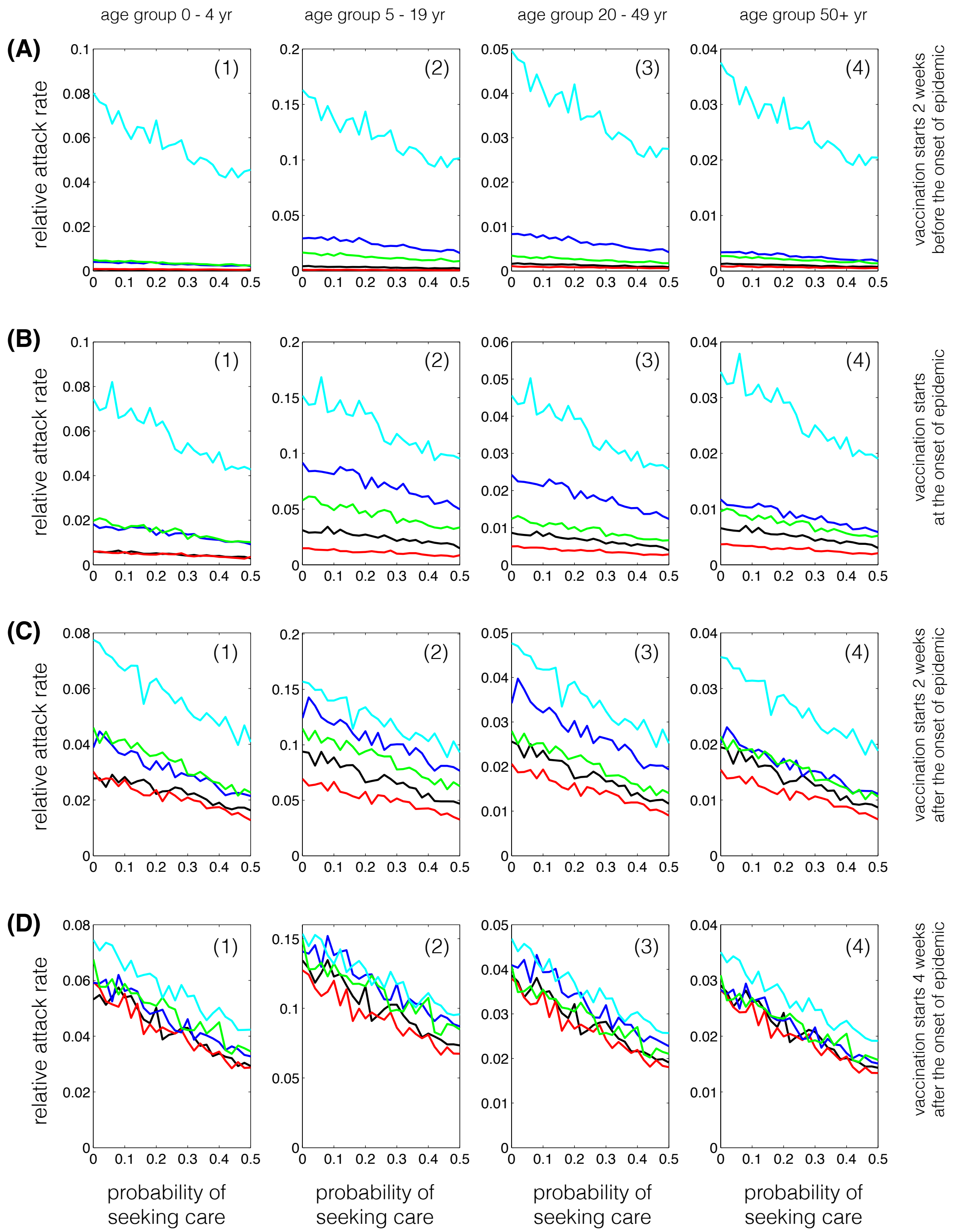

Figure S6. Relative attack rates for single-dose vaccination strategies in urban centre. Curves correspond to the scenarios without vaccination (cyan), morbidity-based vaccination strategy (red), risk-based vaccination strategy (black), outcome-based vaccination strategy (blue), and random vaccination strategy (green).

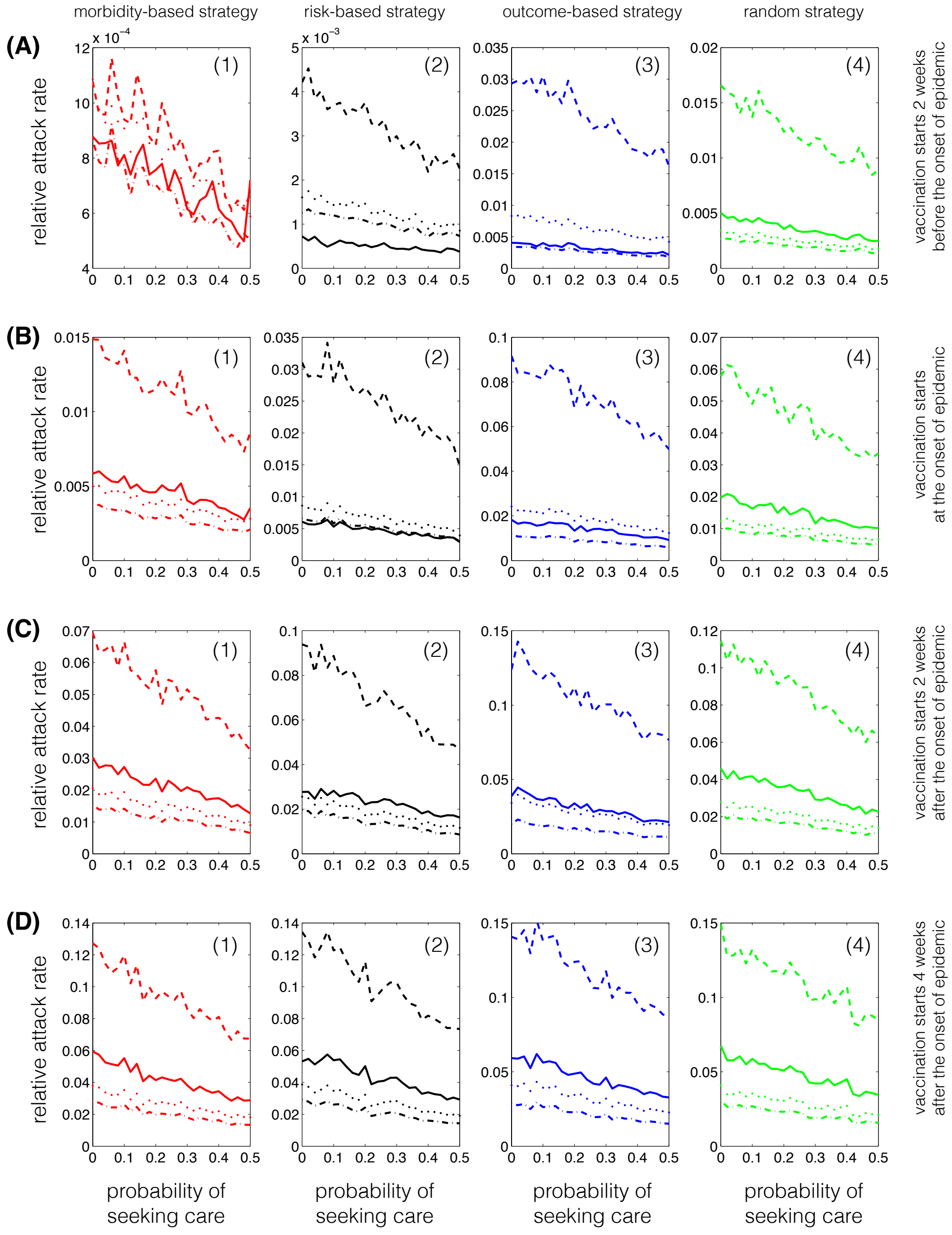

Figure S7. Relative attack rates for single-dose vaccination strategies in urban centre for age groups 0 – 4 (solid curves); 5 – 19 (dashed curves); 20 – 49 (dotted curves); and 50+ (dot-dashed curves). Colours correspond to morbidity-based (red), risk-based (black), outcome-based (blue), and random (green) vaccination strategies.

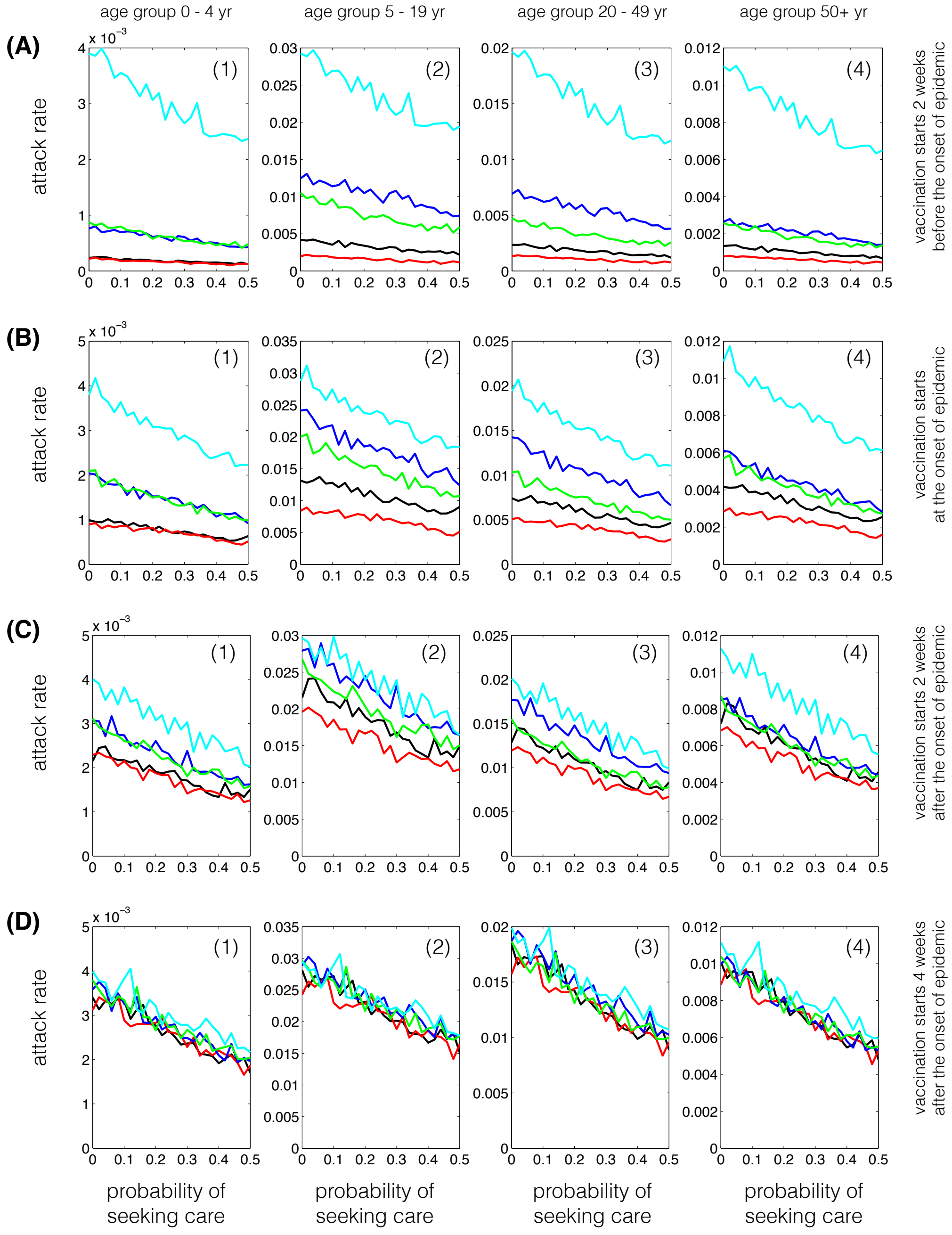

Figure S8. Age-specific attack rates for two-dose vaccination strategies in urban centre. Curves correspond to the scenarios without vaccination (cyan), morbidity-based vaccination strategy (red), risk-based vaccination strategy (black), outcome-based vaccination strategy (blue), and random vaccination strategy (green).

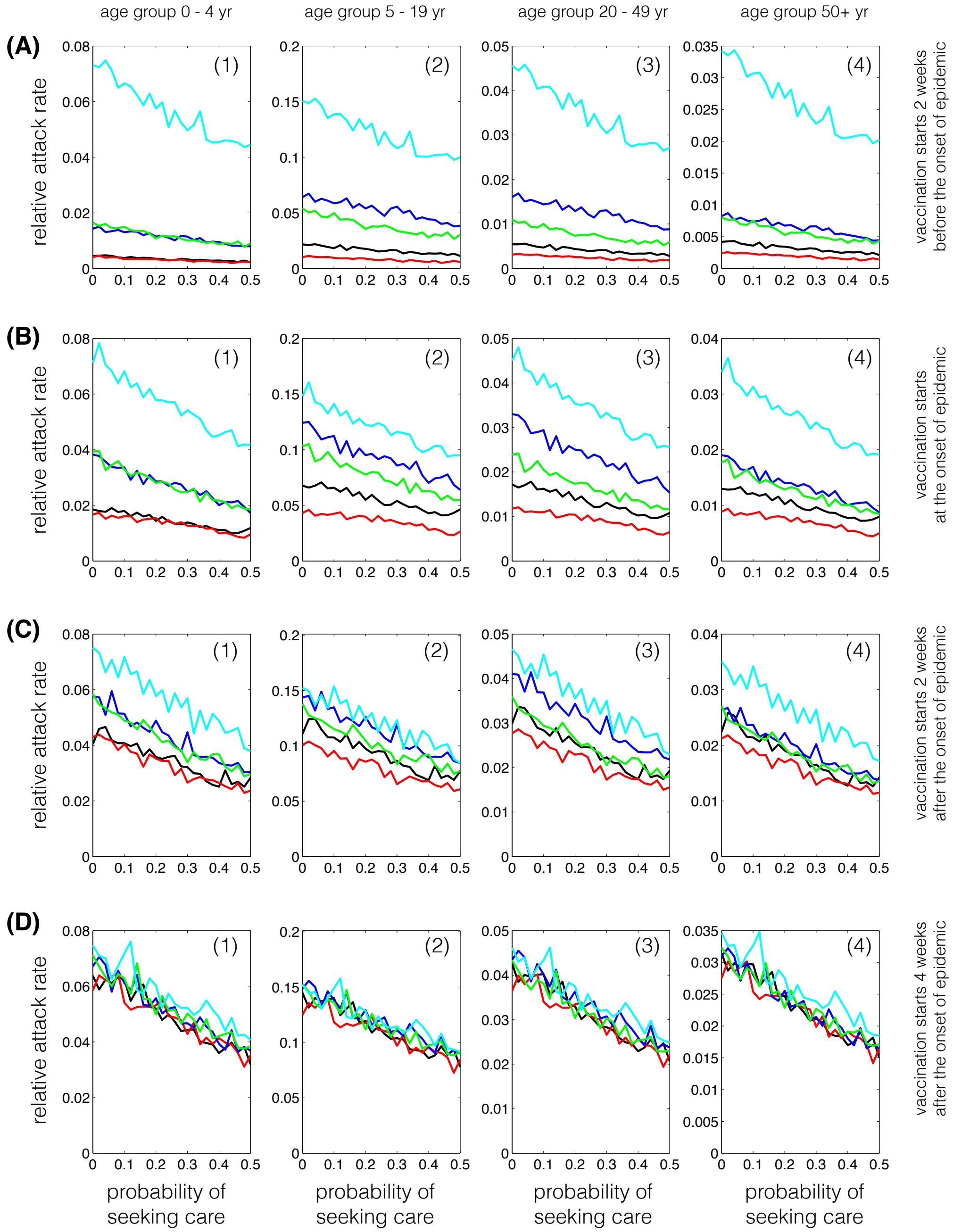

Figure S9. Relative attack rates for two-dose vaccination strategies in urban centre. Curves correspond to the scenarios without vaccination (cyan), morbidity-based vaccination strategy (red), risk-based vaccination strategy (black), outcome-based vaccination strategy (blue), and random vaccination strategy (green).

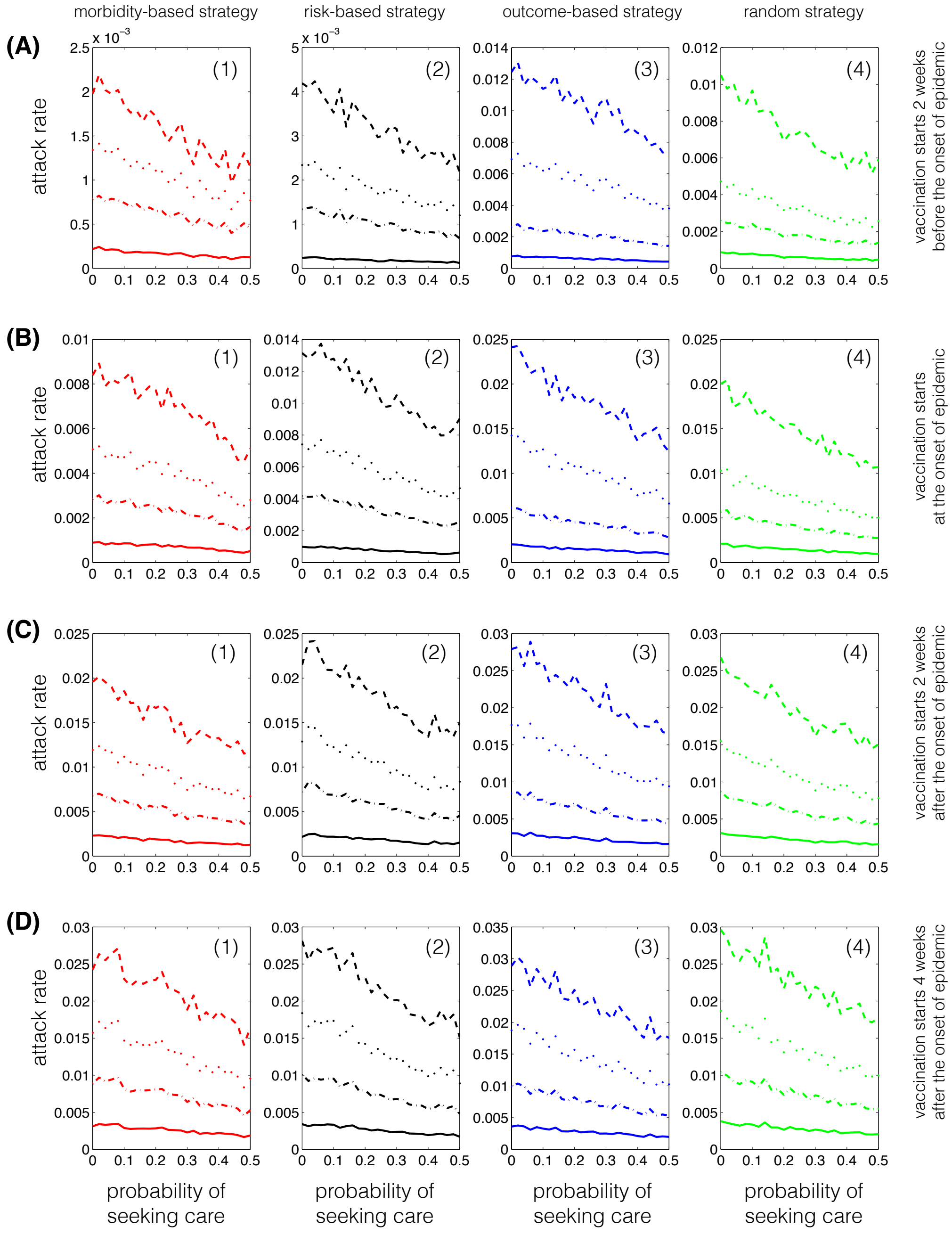

Figure S10. Age-specific attack rates for two-dose vaccination strategies in urban centre for age groups 0 – 4 (solid curves); 5 – 19 (dashed curves); 20 – 49 (dotted curves); and 50+ (dot-dashed curves). Colours correspond to morbidity-based (red), risk-based (black), outcome-based (blue), and random (green) vaccination strategies.

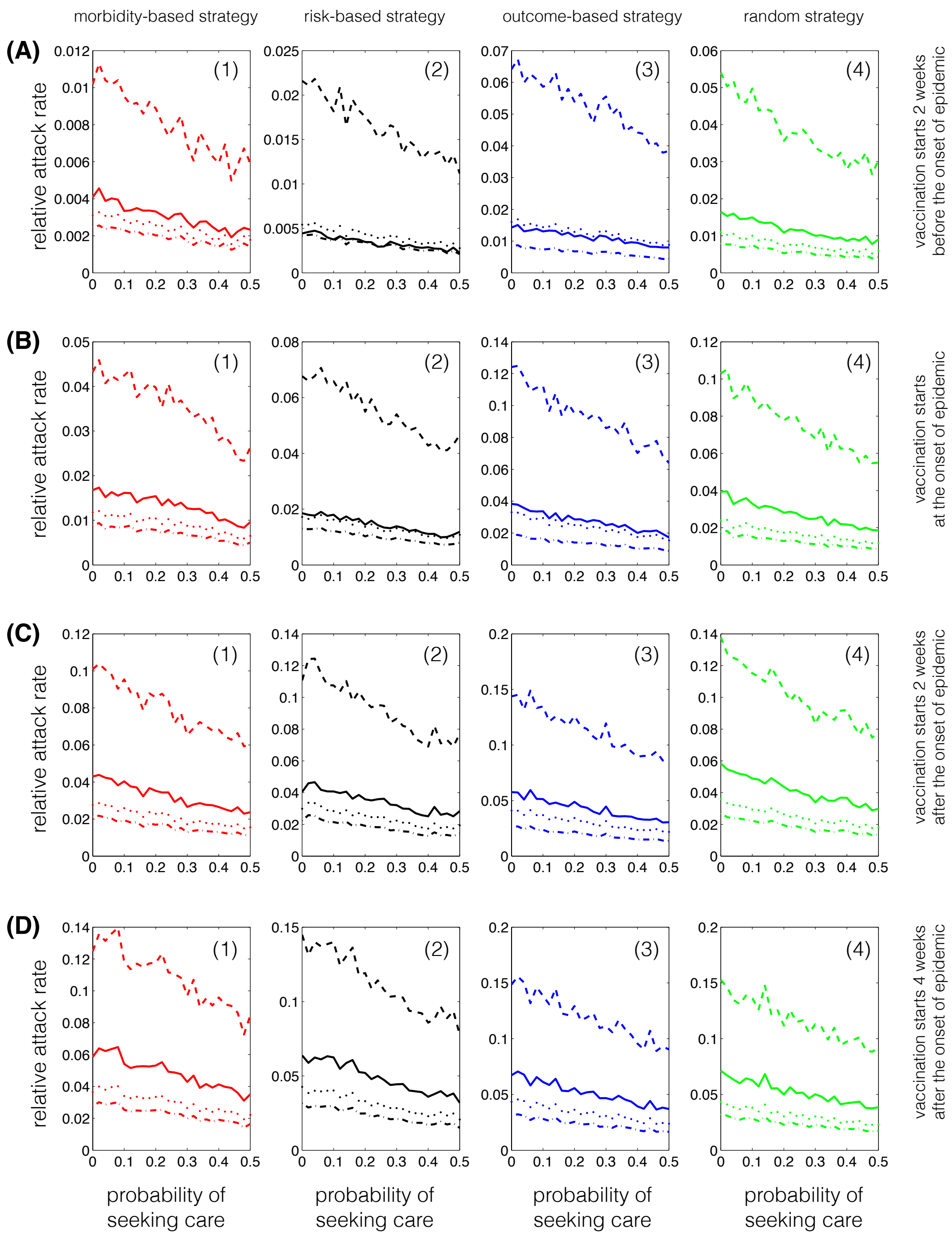

Figure S11. Relative attack rates for two-dose vaccination strategies in urban centre for age groups 0 – 4 (solid curves); 5 – 19 (dashed curves); 20 – 49 (dotted curves); and 50+ (dot-dashed curves). Colours correspond to morbidity-based (red), risk-based (black), outcome-based (blue), and random (green) vaccination strategies.

7. Simulations for shifted demographics (remote community) with $R_0 = 1.6$

Simulations presented in this section correspond to the results presented in the main text for the remote population.

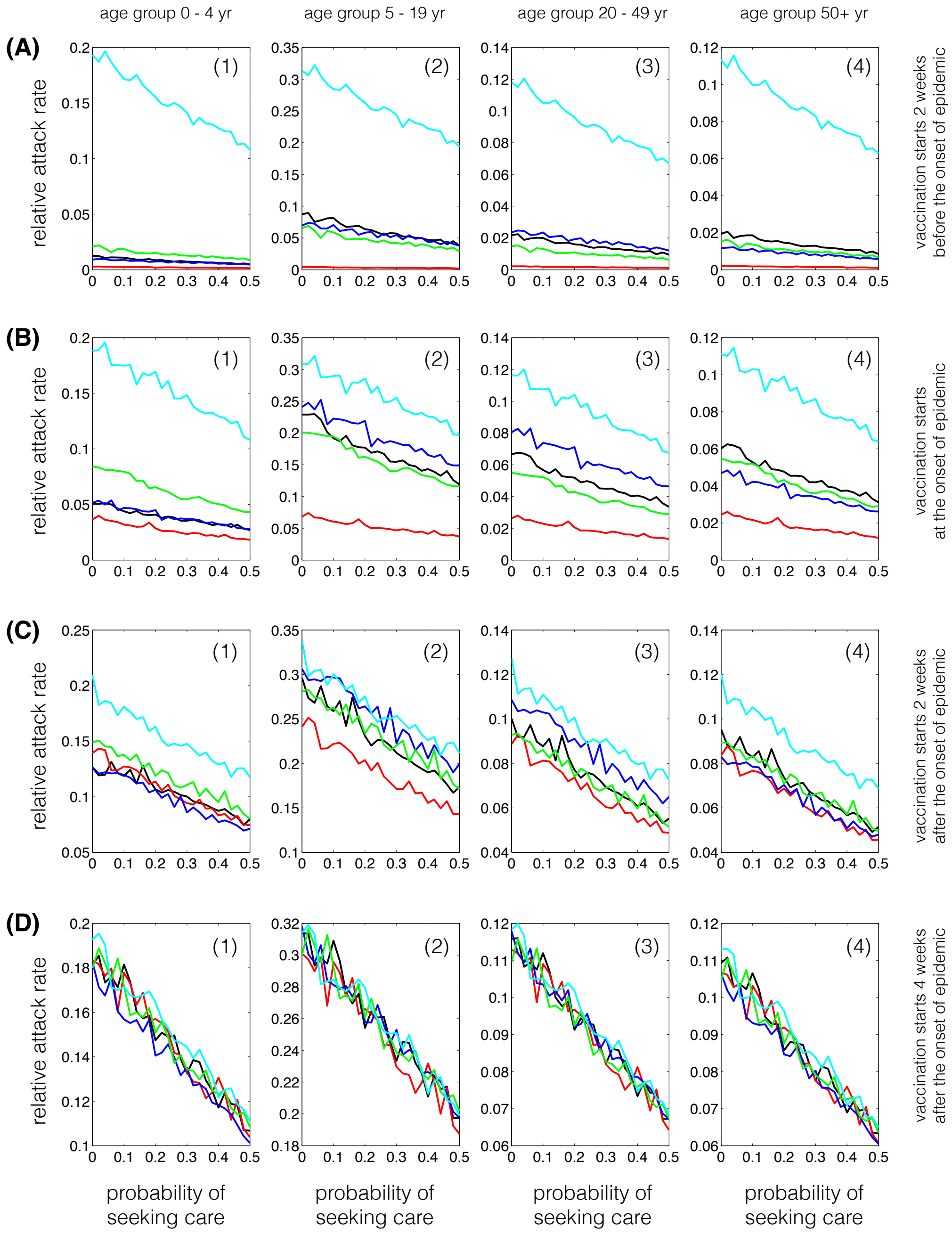

Figure S12. Relative attack rates for single-dose vaccination strategies in shifted demographics (remote community). Curves correspond to the scenarios without vaccination (cyan), morbidity-based vaccination strategy (red), risk-based vaccination strategy (black), outcome-based vaccination strategy (blue), and random vaccination strategy (green).

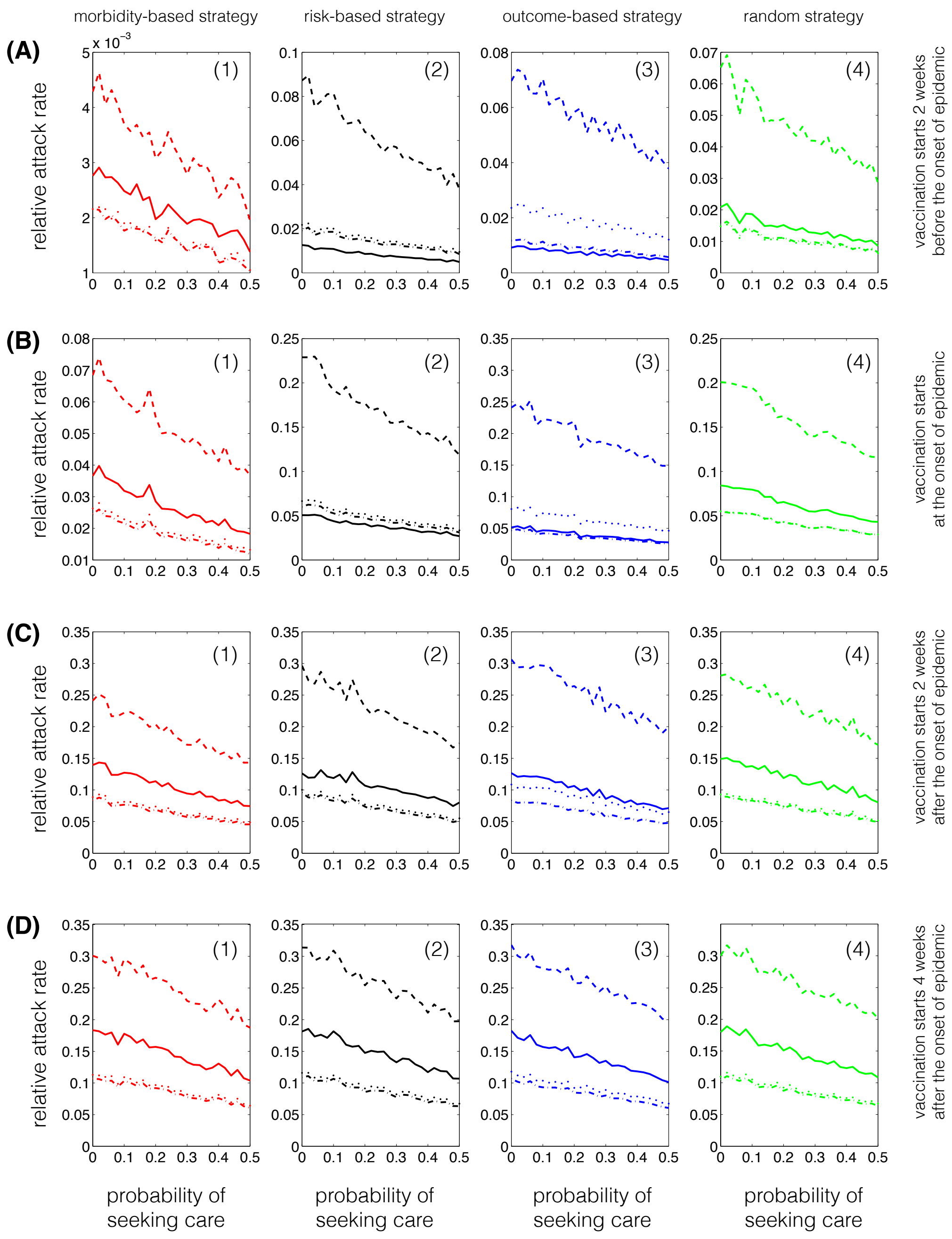

Figure S13. Relative attack rates for single-dose vaccination strategies in shifted demographics (remote community) for age groups 0 – 4 (solid curves); 5 – 19 (dashed curves); 20 – 49 (dotted curves); and 50+ (dot-dashed curves). Colours correspond to morbidity-based (red), risk-based (black), outcome-based (blue), and random (green) vaccination strategies.

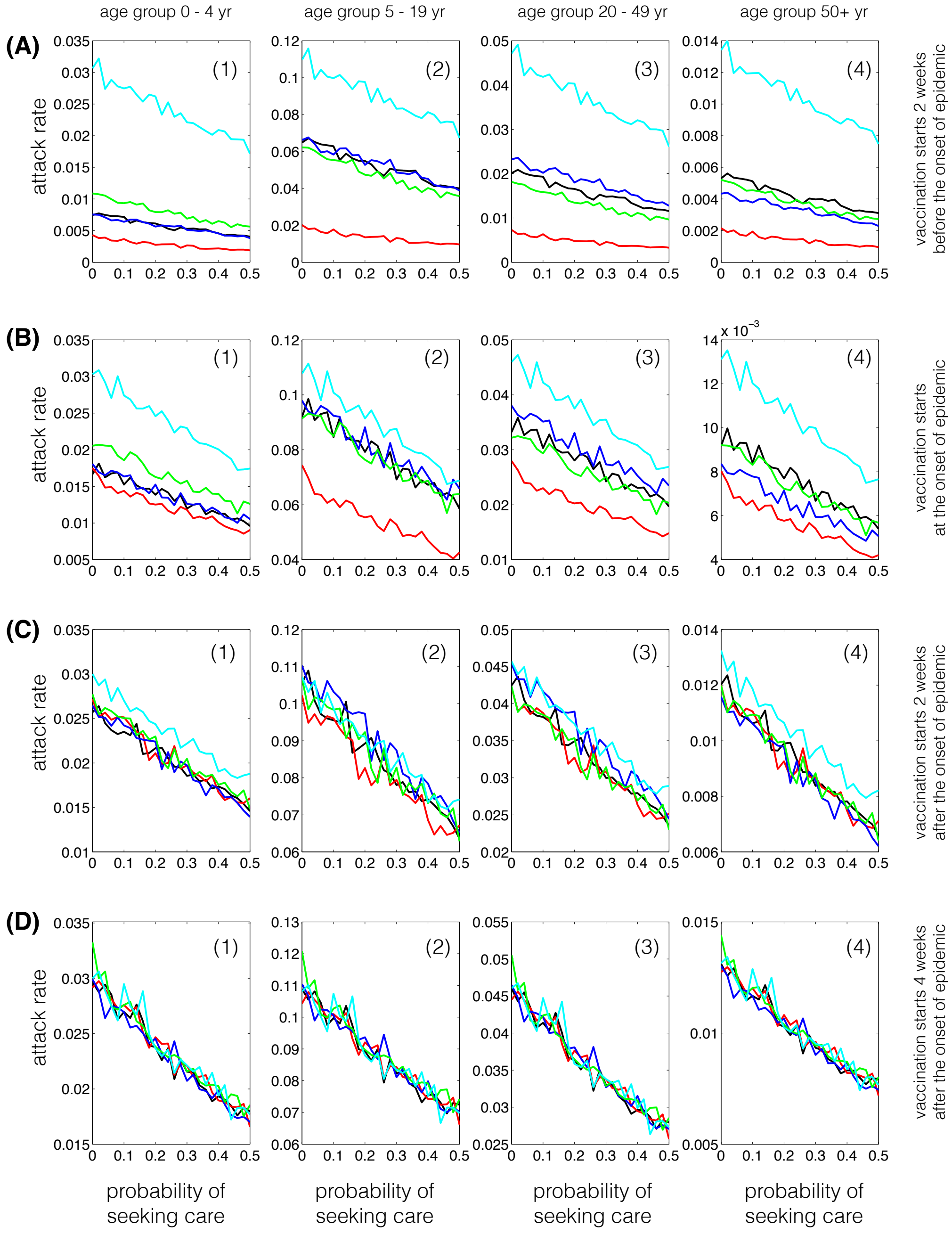

Figure S14. Age-specific attack rates for two-dose vaccination strategies in shifted demographics (remote community). Curves correspond to the scenarios without vaccination (cyan), morbidity-based vaccination strategy (red), risk-based vaccination strategy (black), outcome-based vaccination strategy (blue), and random vaccination strategy (green).

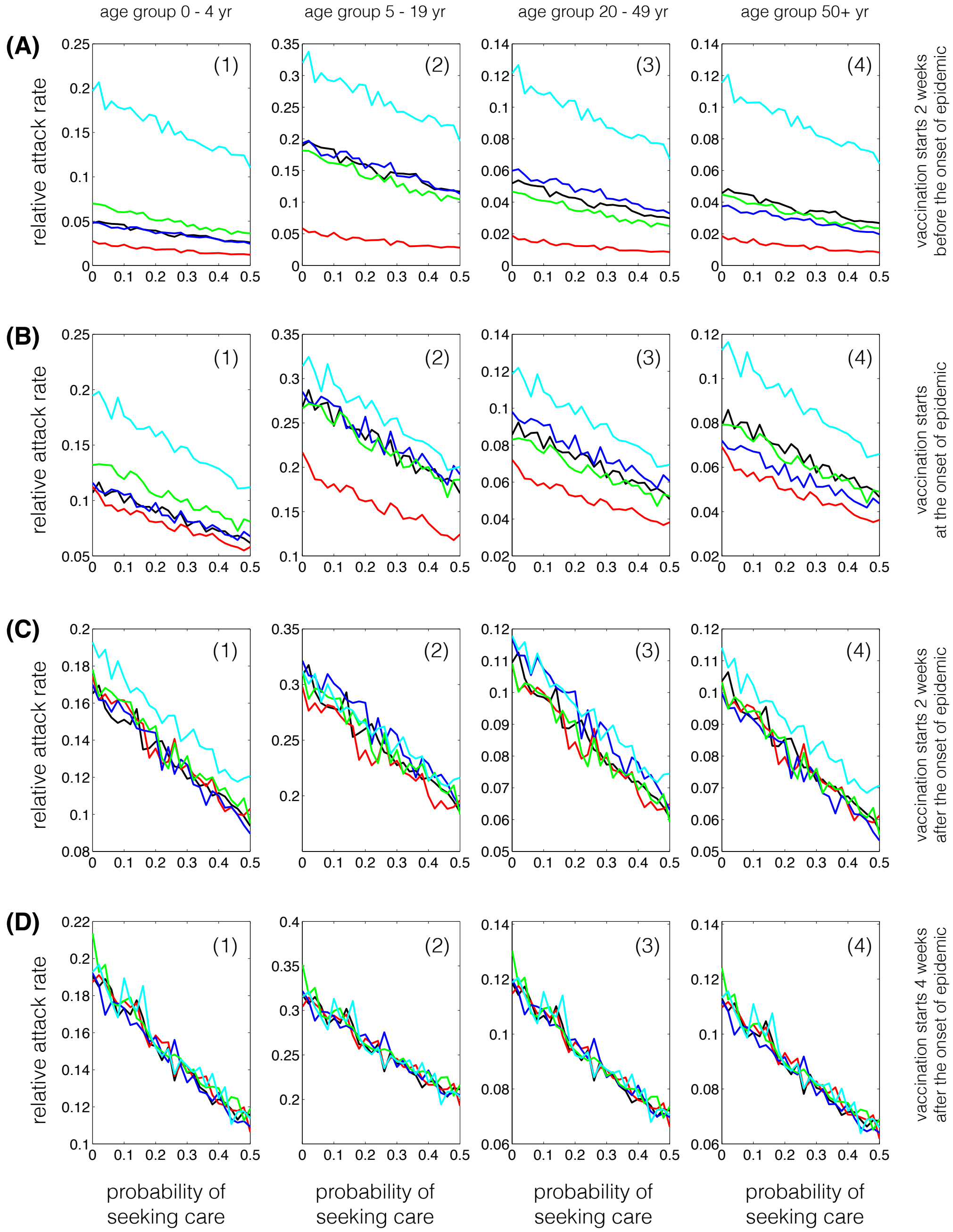

Figure S15. Relative attack rates for two-dose vaccination strategies in shifted demographics (remote community). Curves correspond to the scenarios without vaccination (cyan), morbidity-based vaccination strategy (red), risk-based vaccination strategy (black), outcome-based vaccination strategy (blue), and random vaccination strategy (green).

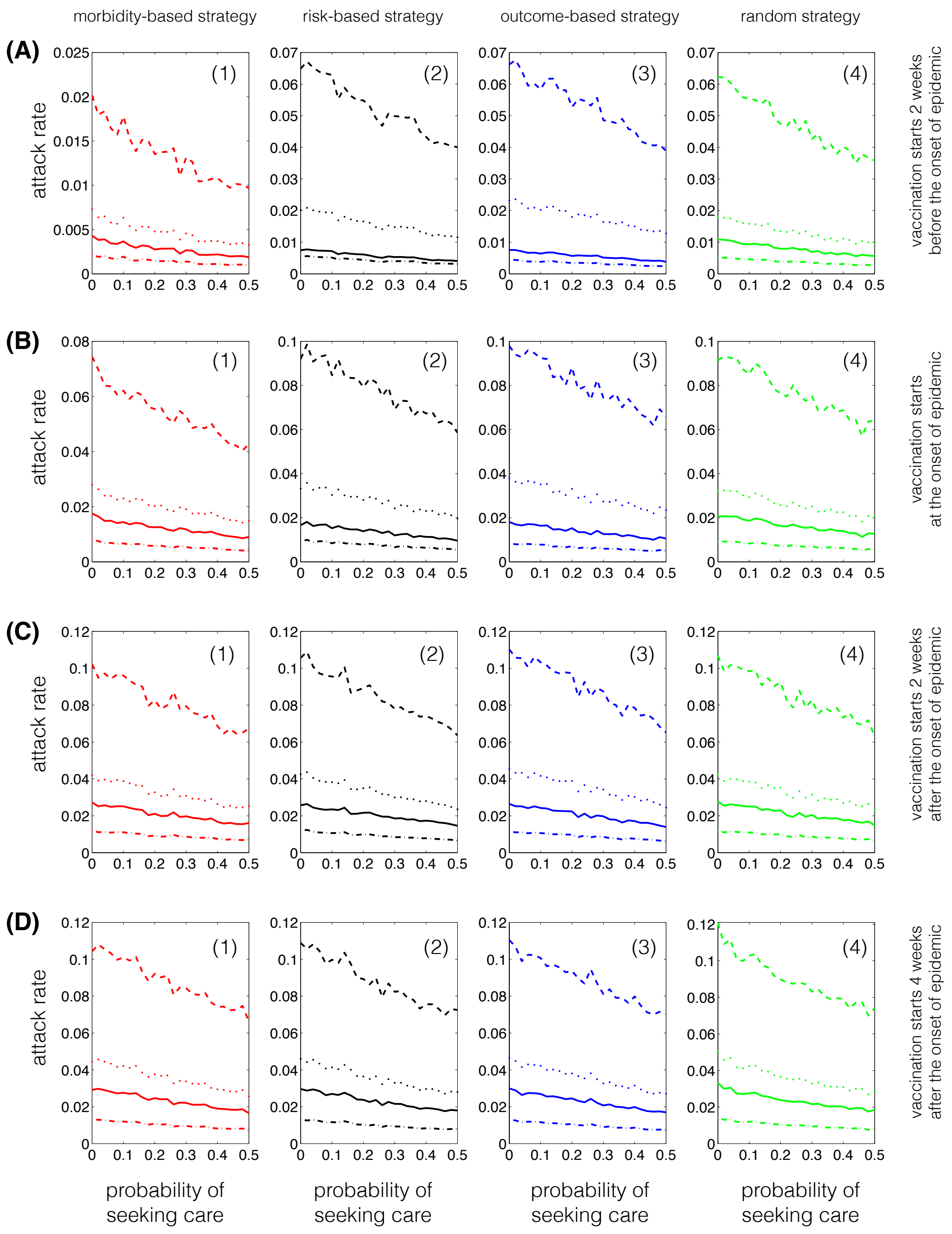

Figure S16. Age-specific attack rates for two-dose vaccination strategies in shifted demographics (remote community) for age groups 0 – 4 (solid curves); 5 – 19 (dashed curves); 20 – 49 (dotted curves); and 50+ (dot-dashed curves). Colours correspond to morbidity-based (red), risk-based (black), outcome-based (blue), and random (green) vaccination strategies.

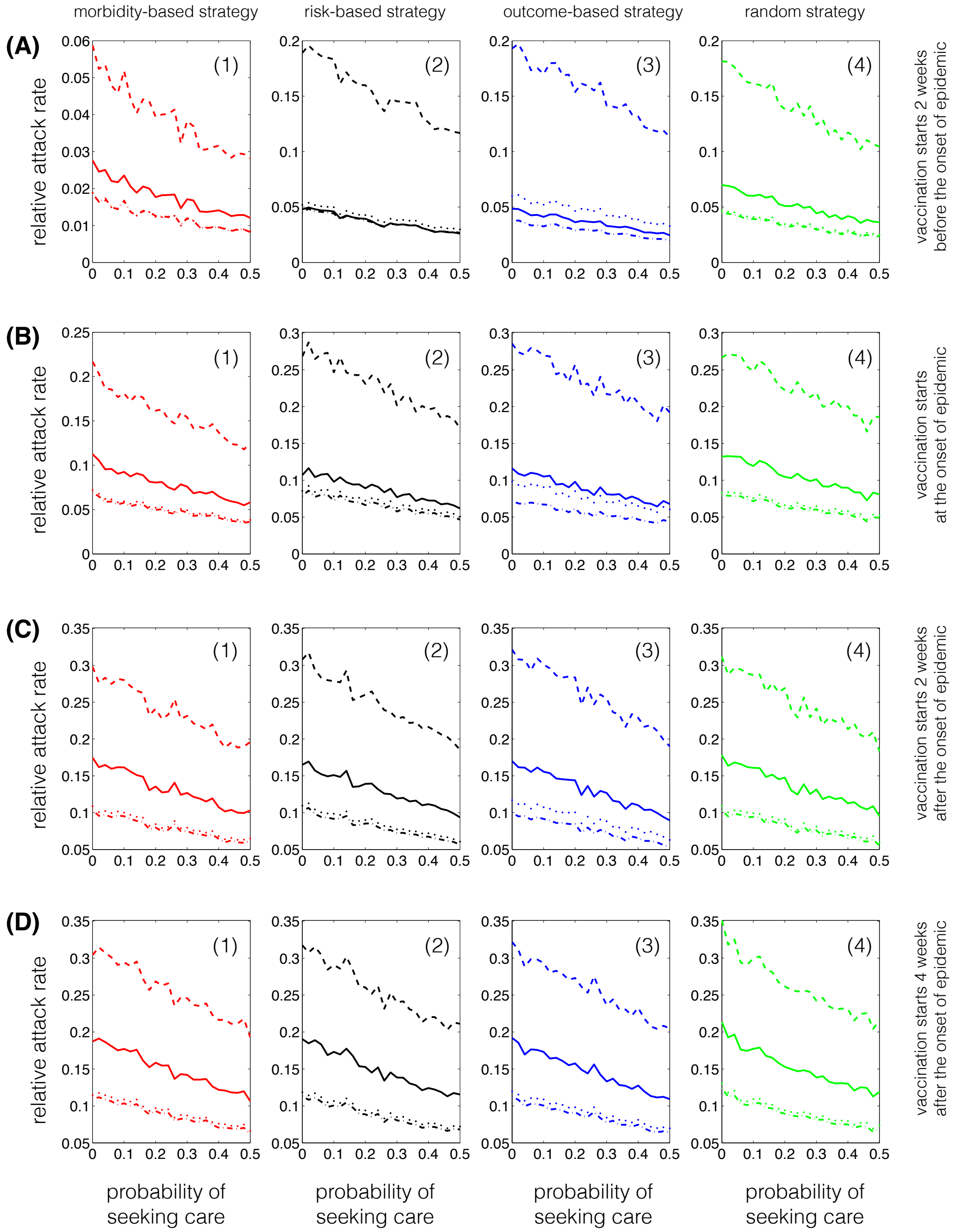

Figure S17. Relative attack rates for two-dose vaccination strategies in shifted demographics (remote community) for age groups 0 – 4 (solid curves); 5 – 19 (dashed curves); 20 – 49 (dotted curves); and 50+ (dot-dashed curves). Colours correspond to morbidity-based (red), risk-based (black), outcome-based (blue), and random (green) vaccination strategies.

8. Percentage reduction of hospitalization for two-dose vaccination strategies

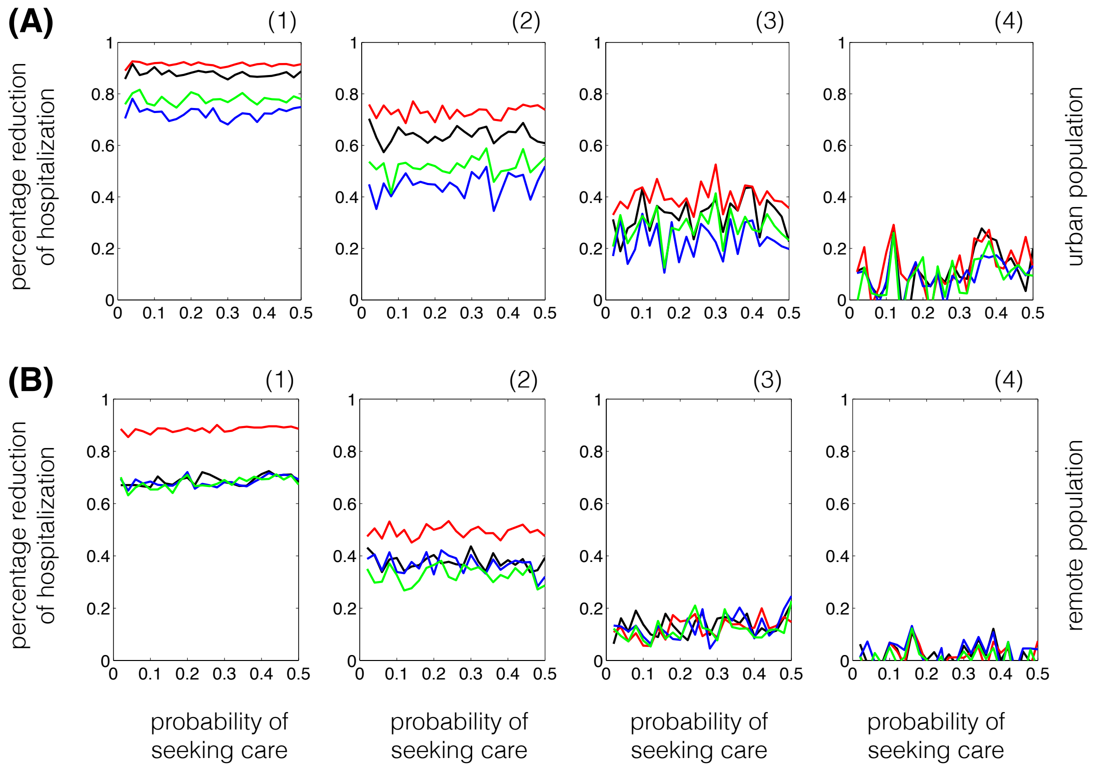

Figure S18. Percentage reduction of hospitalization for different vaccination strategies compared to the scenario without vaccination. Panel (A) and (B) correspond to two-dose vaccination strategies in urban ($R_0 = 1.3$) and remote ($R_0 = 1.6$) populations, respectively, with the start of vaccination (1): two weeks before the onset of epidemic; (2): at the onset of epidemic; (3) two weeks after the onset of epidemic; and (4): four weeks after the onset of epidemic. Colours correspond to the morbidity-based (red), risk-based (black), outcome-based (blue), and random (green) vaccination strategies. The horizontal axis represents the fraction of symptomatically infected individuals who seek care during symptomatic infection.

9. Percentage reduction of hospitalization for single-dose vaccination strategies

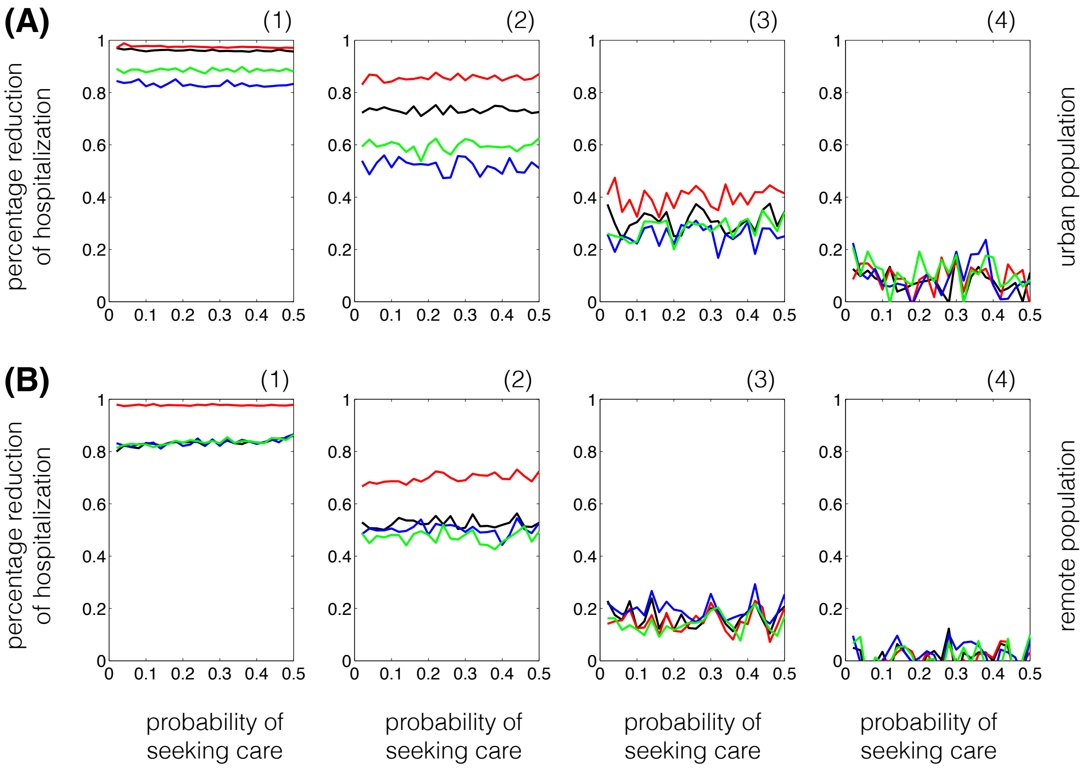

Figure S19. Percentage reduction of hospitalization for different age groups in outcome-based strategy compared to the scenario without vaccination. Panel (A) and (B) correspond to a single-dose vaccination strategy in urban ($R_0 = 1.7$) and remote ($R_0 = 2.0$) populations, respectively, with the start of vaccination (1): two weeks before the onset of epidemic; (2): at the onset of epidemic; (3) two weeks after the onset of epidemic; and (4): four weeks after the onset of epidemic. Colours correspond to the 0 – 4 (black), 5 – 19 (red), 20 – 49 (blue), and 50+ (green) years age groups. The horizontal axis represents the fraction of symptomatically infected individuals who seek care during symptomatic infection.

10. Percentage reduction of hospitalization for two-dose vaccination strategies

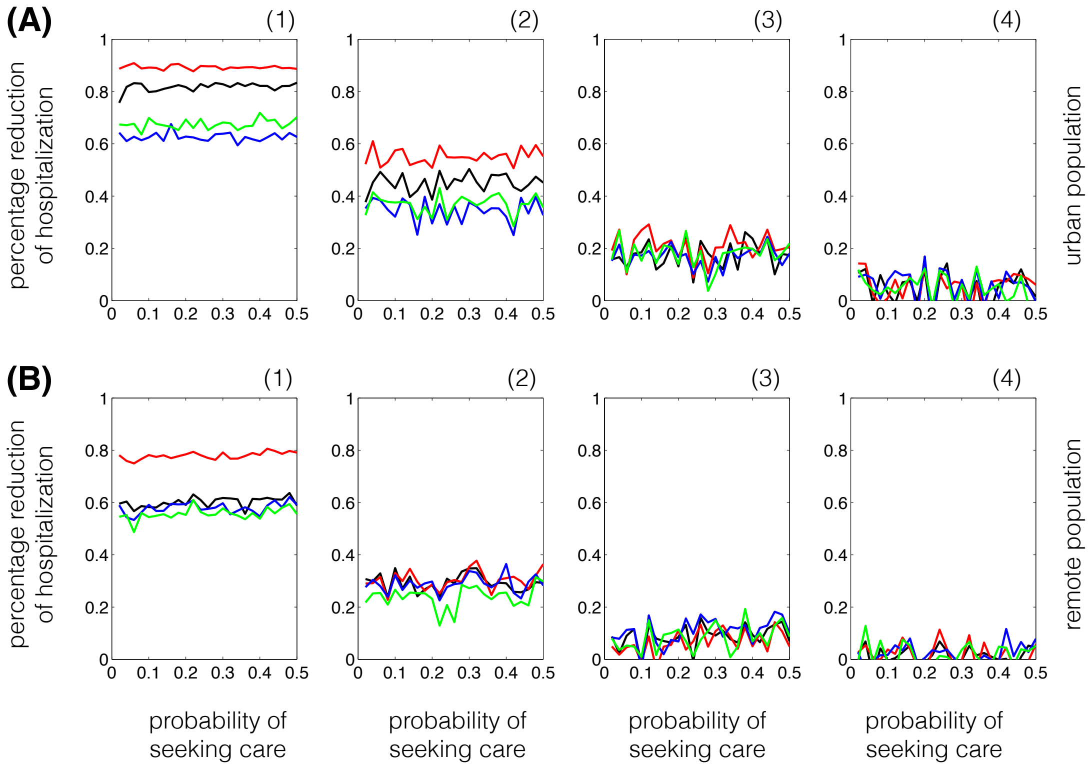

Figure S20. Percentage reduction of hospitalization for different vaccination strategies compared to the scenario without vaccination. Panel (A) and (B) correspond to two-dose vaccination strategies in urban ($R_0 = 1.7$) and remote ($R_0 = 2.0$) populations, respectively, with the start of vaccination (1): two weeks before the onset of epidemic; (2): at the onset of epidemic; (3) two weeks after the onset of epidemic; and (4): four weeks after the onset of epidemic. Colours correspond to the morbidity-based (red), risk-based (black), outcome-based (blue), and random (green) vaccination strategies. The horizontal axis represents the fraction of symptomatically infected individuals who seek care during symptomatic infection.

11. Simulations for urban centre with $R_0 = 1.7$

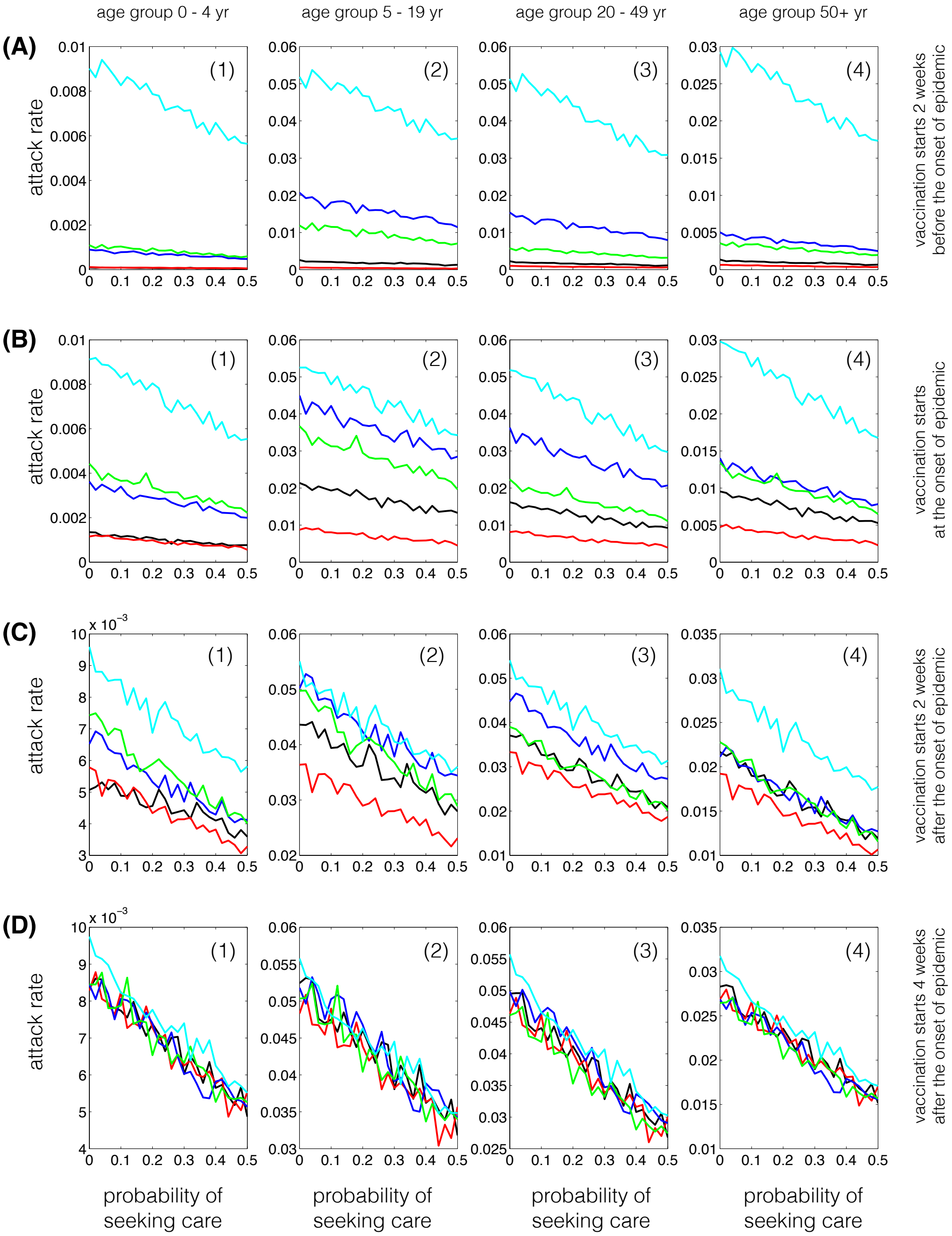

Figure S21. Age-specific attack rates for single-dose vaccination strategies in urban centre. Curves correspond to the scenarios without vaccination (cyan), morbidity-based vaccination strategy (red), risk-based vaccination strategy (black), outcome-based vaccination strategy (blue), and random vaccination strategy (green).

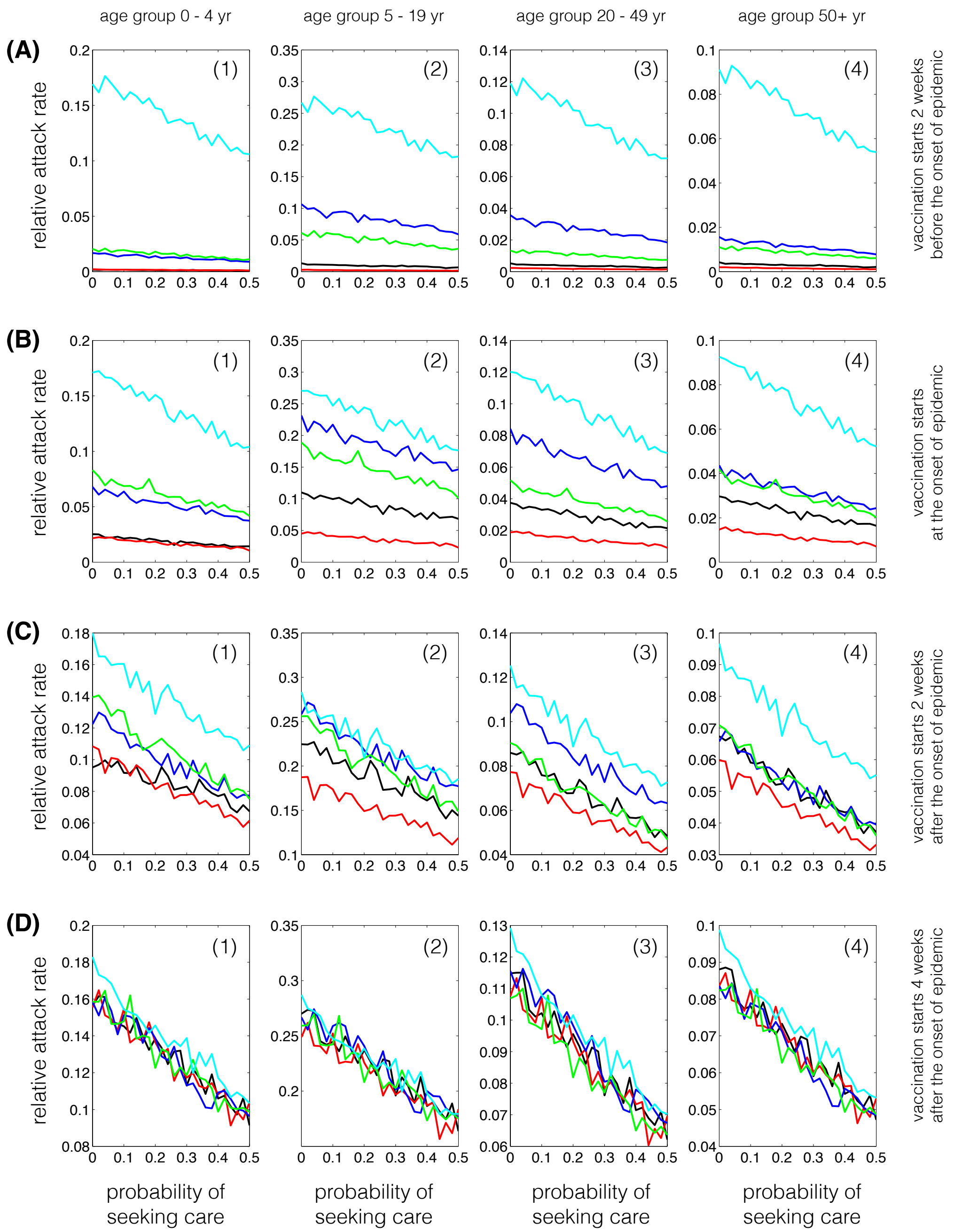

Figure S22. Relative attack rates for single-dose vaccination strategies in urban centre. Curves correspond to the scenarios without vaccination (cyan), morbidity-based vaccination strategy (red), risk-based vaccination strategy (black), outcome-based vaccination strategy (blue), and random vaccination strategy (green).

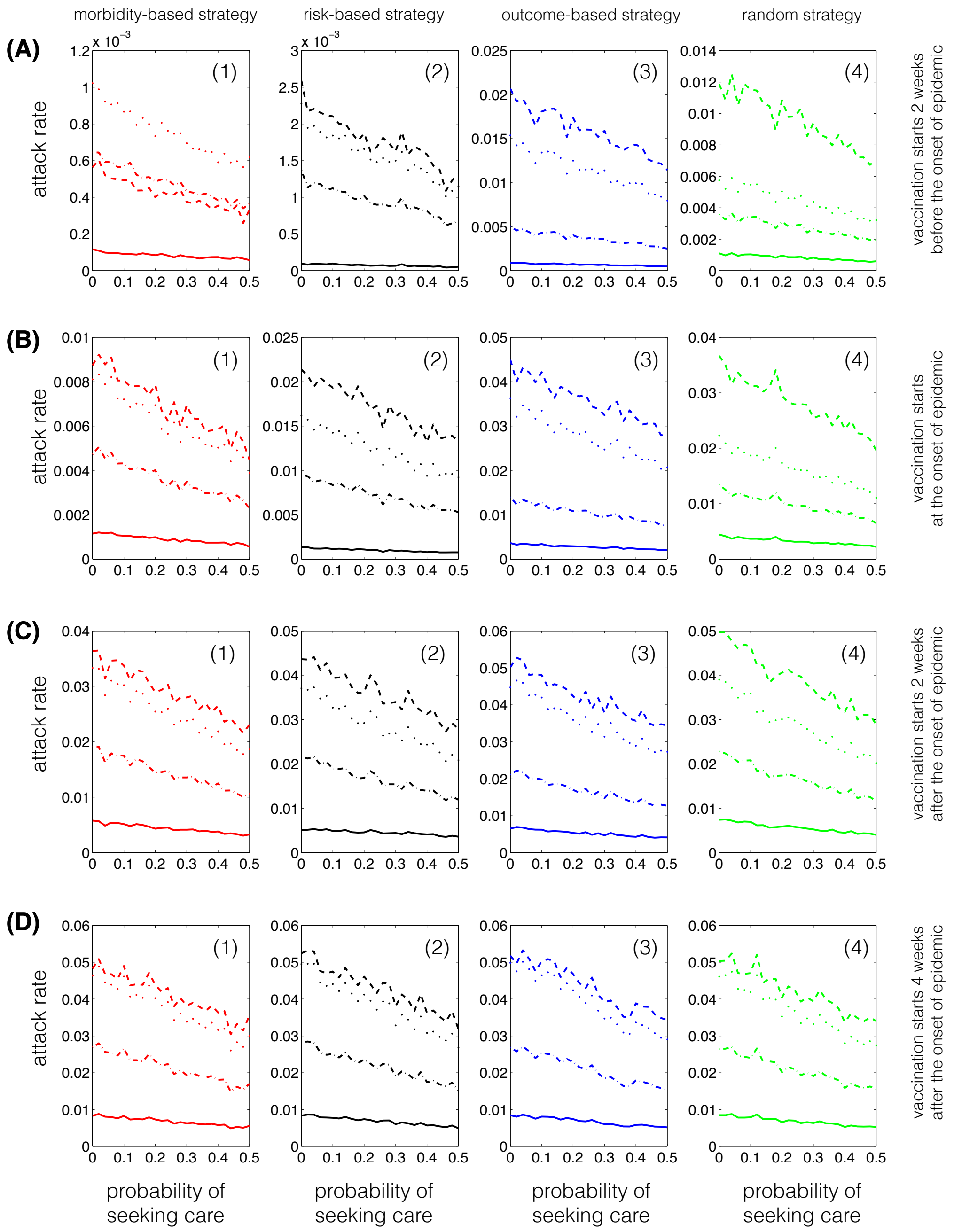

Figure S23. Age-specific attack rates for single-dose vaccination strategies in urban centre for age groups 0 – 4 (solid curves); 5 – 19 (dashed curves); 20 – 49 (dotted curves); and 50+ (dot-dashed curves). Colours correspond to morbidity-based (red), risk-based (black), outcome-based (blue), and random (green) vaccination strategies.

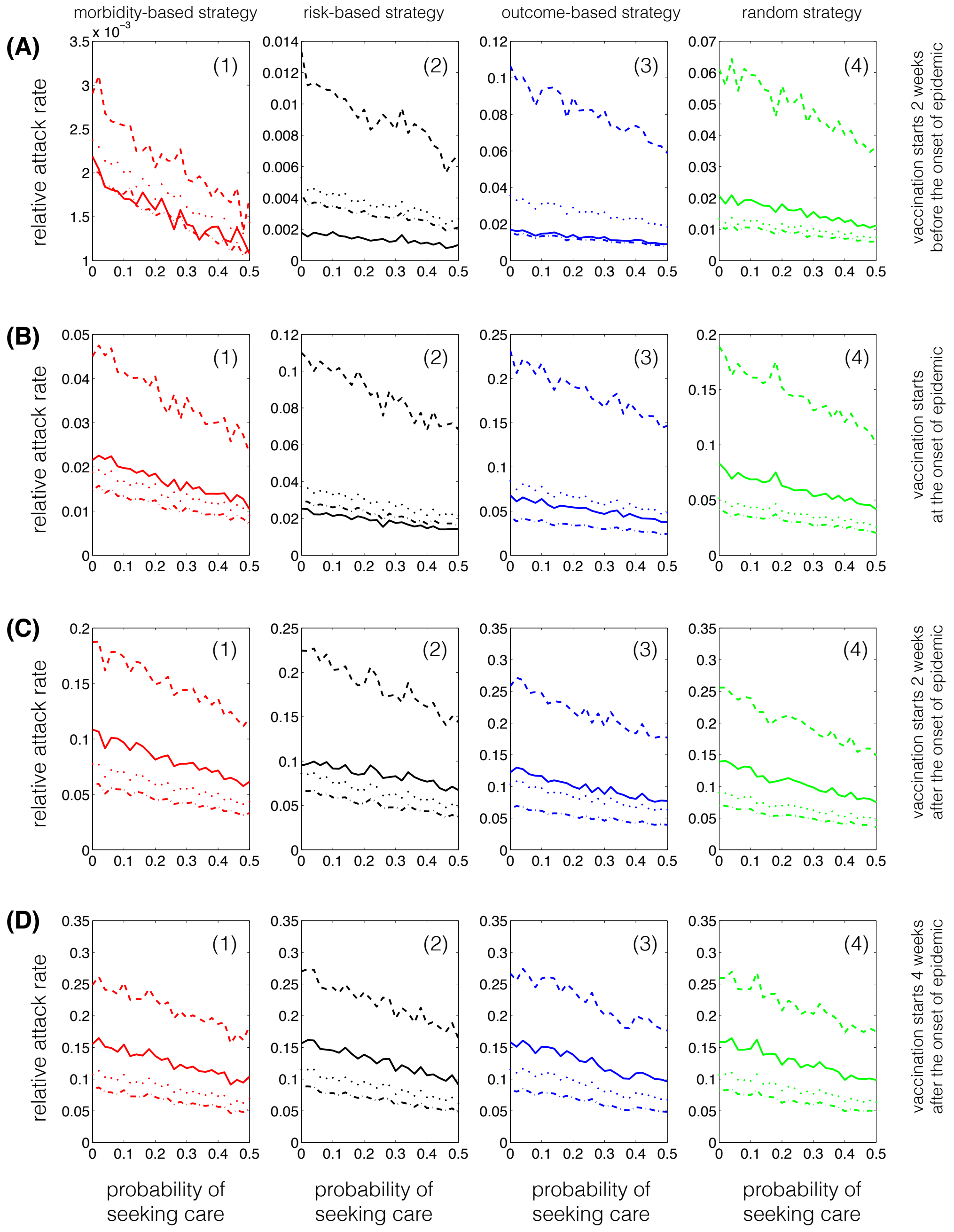

Figure S24. Relative attack rates for single-dose vaccination strategies in urban centre for age groups 0 – 4 (solid curves); 5 – 19 (dashed curves); 20 – 49 (dotted curves); and 50+ (dot-dashed curves). Colours correspond to morbidity-based (red), risk-based (black), outcome-based (blue), and random (green) vaccination strategies.

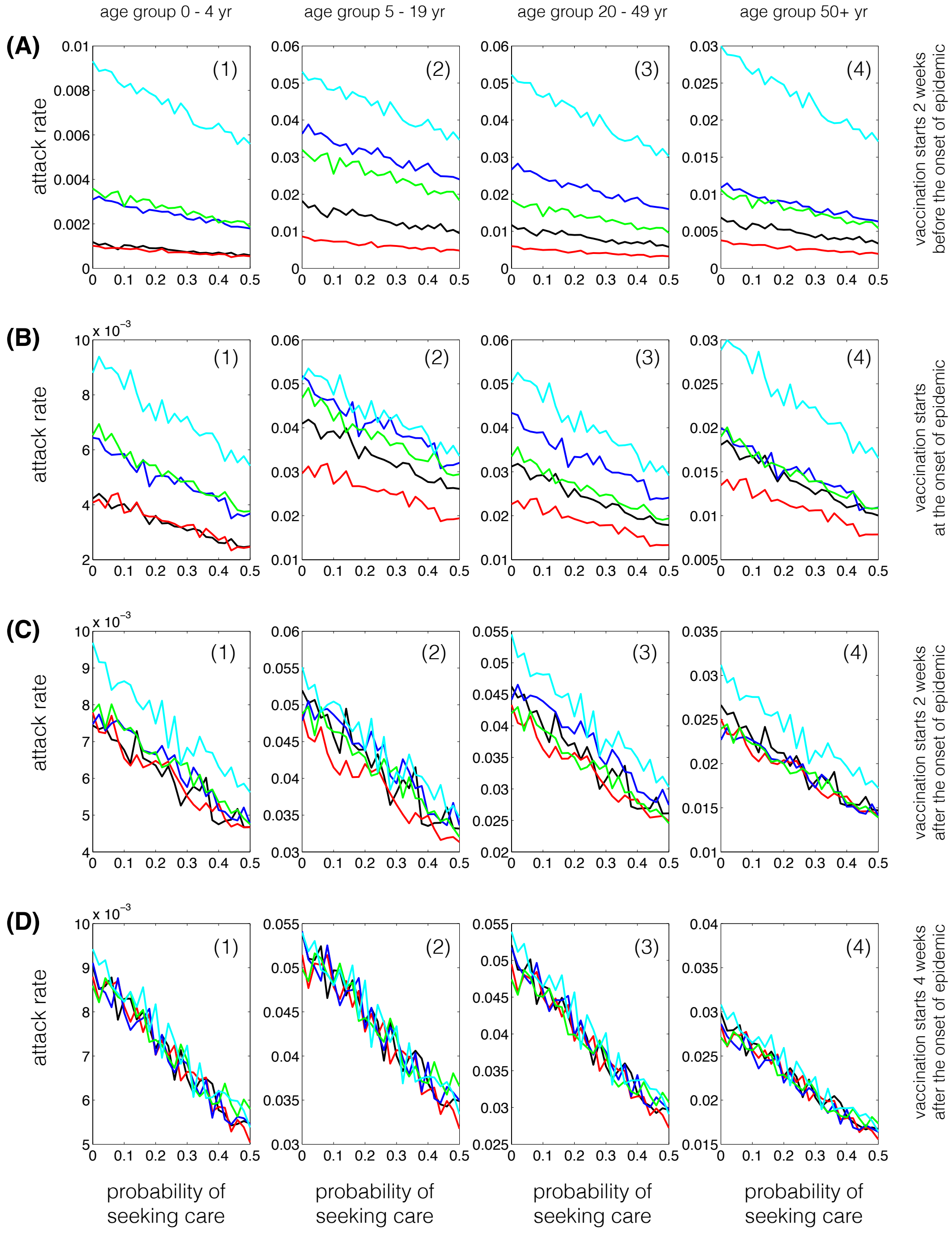

Figure S25. Age-specific attack rates for two-dose vaccination strategies in urban centre. Curves correspond to the scenarios without vaccination (cyan), morbidity-based vaccination strategy (red), risk-based vaccination strategy (black), outcome-based vaccination strategy (blue), and random vaccination strategy (green).

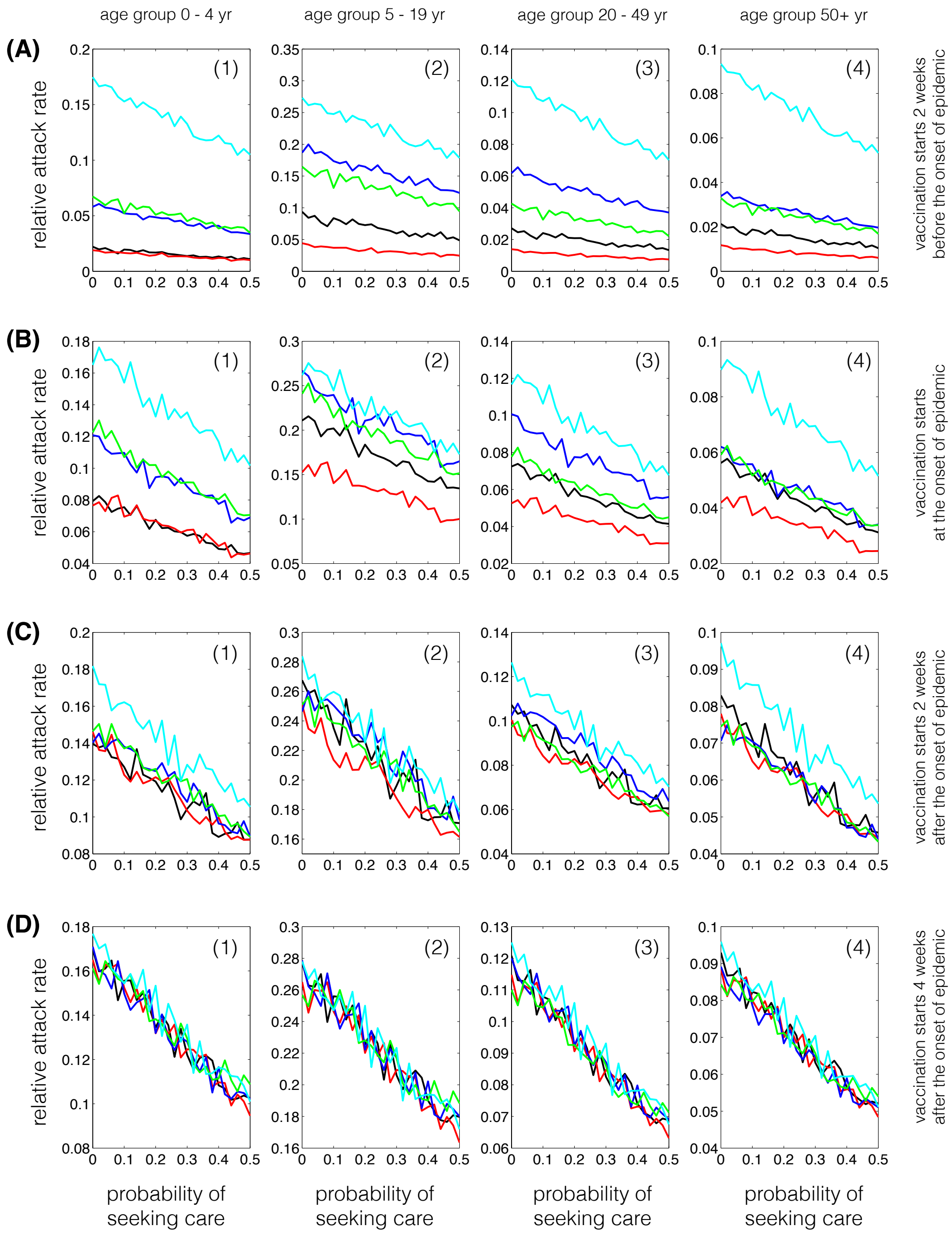

Figure S26. Relative attack rates for two-dose vaccination strategies in urban centre. Curves correspond to the scenarios without vaccination (cyan), morbidity-based vaccination strategy (red), risk-based vaccination strategy (black), outcome-based vaccination strategy (blue), and random vaccination strategy (green).

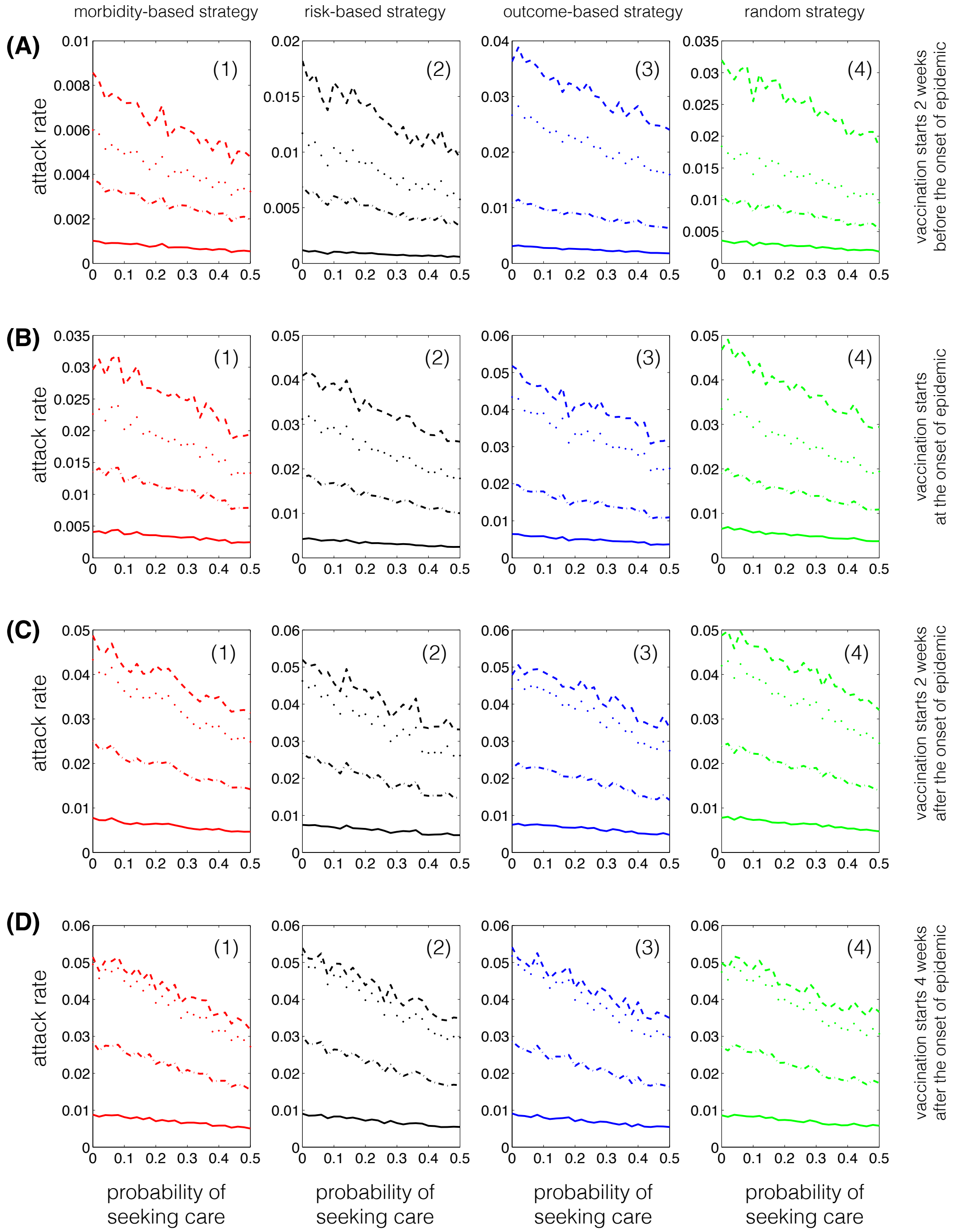

Figure S27. Age-specific attack rates for two-dose vaccination strategies in urban centre for age groups 0 – 4 (solid curves); 5 – 19 (dashed curves); 20 – 49 (dotted curves); and 50+ (dot-dashed curves). Colours correspond to morbidity-based (red), risk-based (black), outcome-based (blue), and random (green) vaccination strategies.

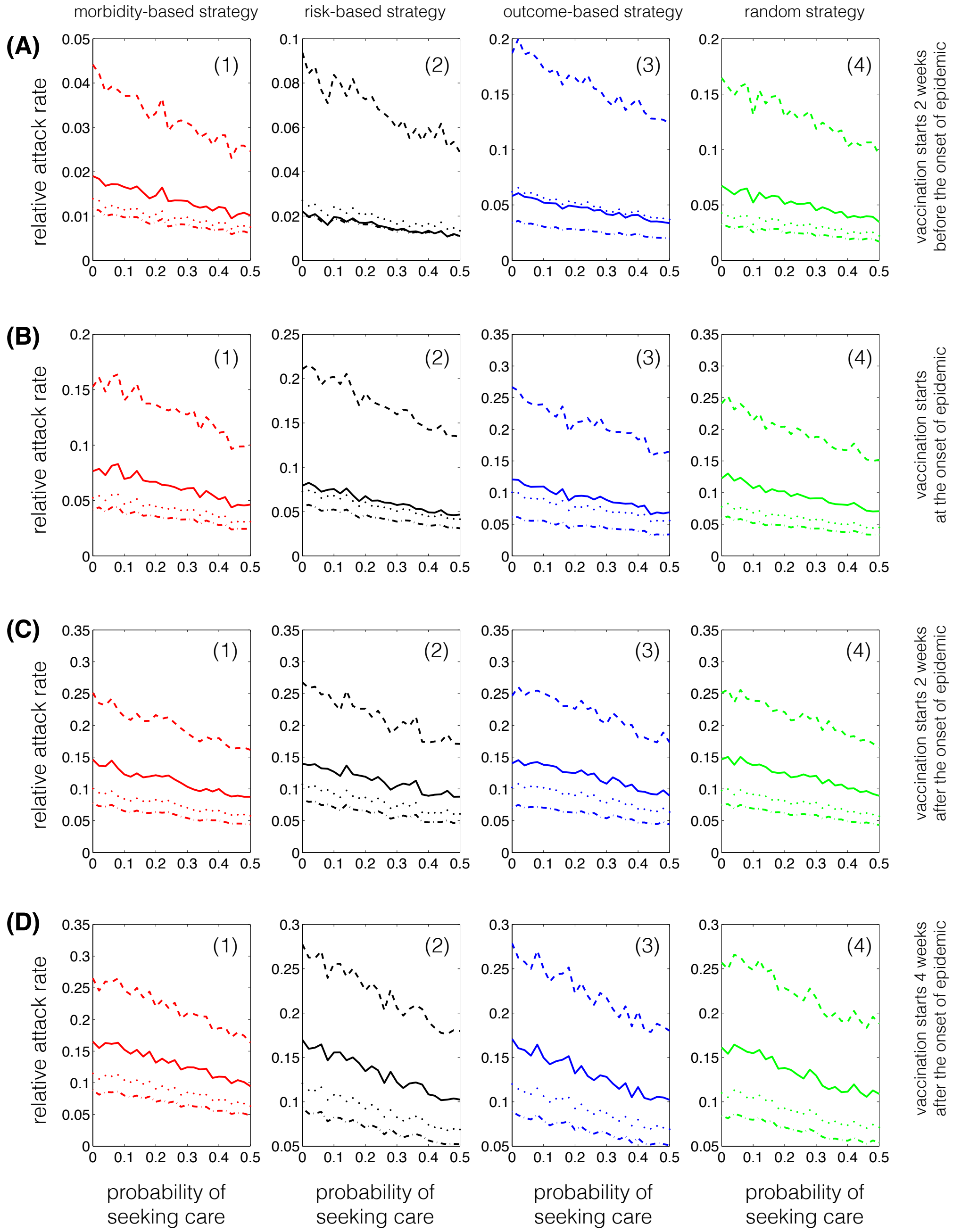

Figure S28. Relative attack rates for two-dose vaccination strategies in urban centre for age groups 0 – 4 (solid curves); 5 – 19 (dashed curves); 20 – 49 (dotted curves); and 50+ (dot-dashed curves). Colours correspond to morbidity-based (red), risk-based (black), outcome-based (blue), and random (green) vaccination strategies.

12. Simulations for shifted demographics (remote community) with $R_0 = 2$

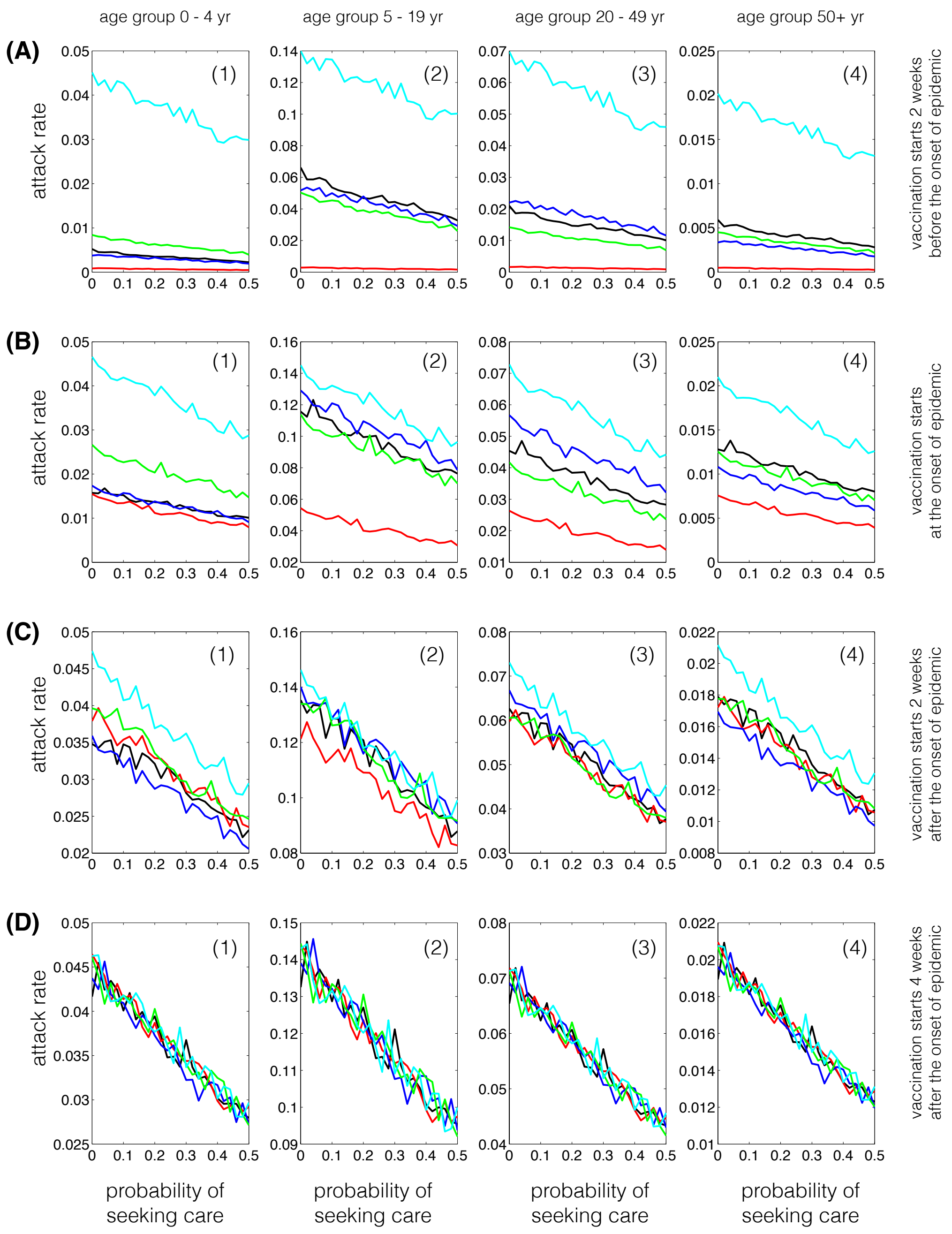

Figure S29. Age-specific attack rates for single-dose vaccination strategies in shifted demographics (remote community). Curves correspond to the scenarios without vaccination (cyan), morbidity-based vaccination strategy (red), risk-based vaccination strategy (black), outcome-based vaccination strategy (blue), and random vaccination strategy (green).

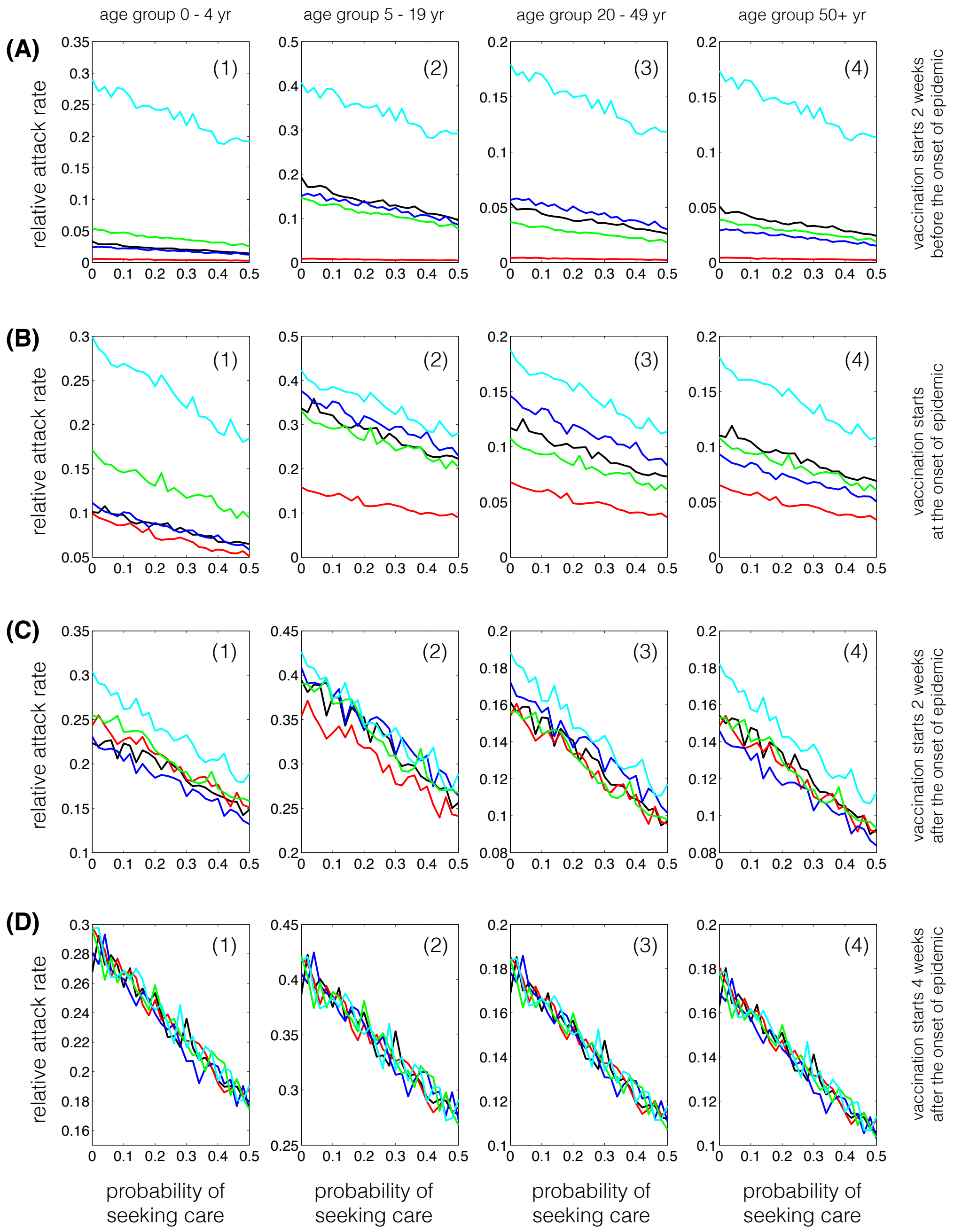

Figure S30. Relative attack rates for single-dose vaccination strategies in shifted demographics (remote community). Curves correspond to the scenarios without vaccination (cyan), morbidity-based vaccination strategy (red), risk-based vaccination strategy (black), outcome-based vaccination strategy (blue), and random vaccination strategy (green).

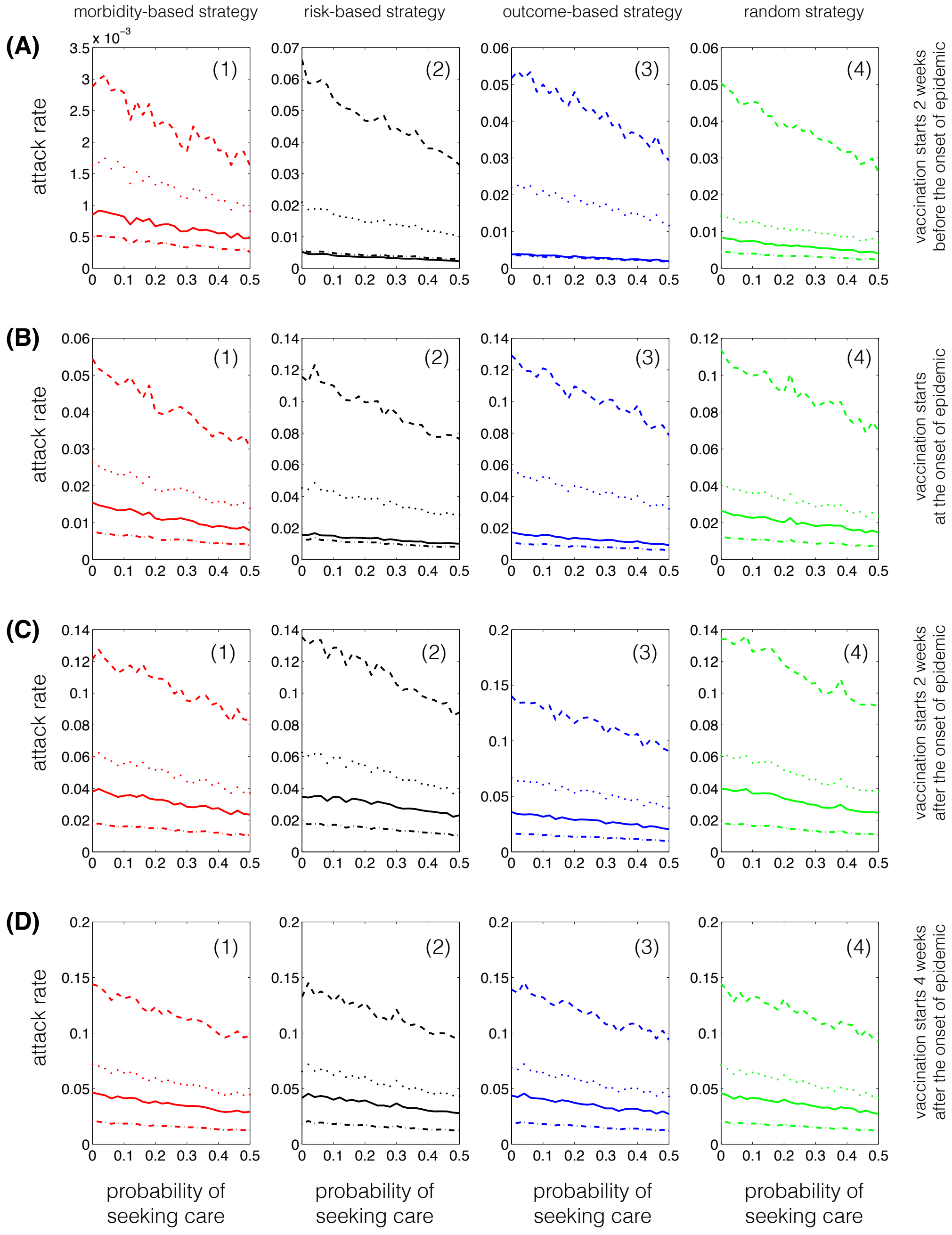

Figure S31. Age-specific attack rates for single-dose vaccination strategies in shifted demographics (remote community) for age groups 0 – 4 (solid curves); 5 – 19 (dashed curves); 20 – 49 (dotted curves); and 50+ (dot-dashed curves). Colours correspond to morbidity-based (red), risk-based (black), outcome-based (blue), and random (green) vaccination strategies.

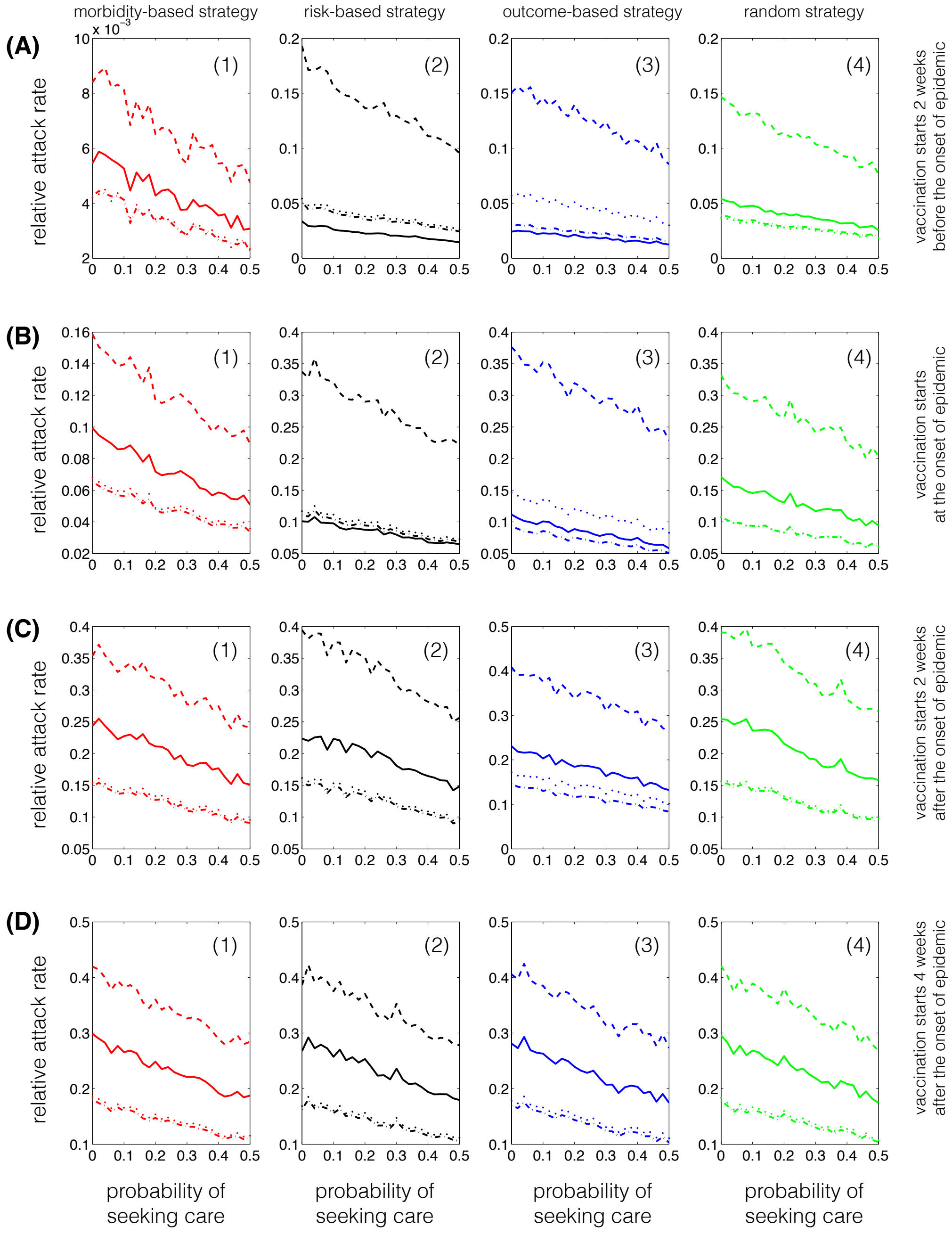

Figure S32. Relative attack rates for single-dose vaccination strategies in shifted demographics (remote community) for age groups 0 – 4 (solid curves); 5 – 19 (dashed curves); 20 – 49 (dotted curves); and 50+ (dot-dashed curves). Colours correspond to morbidity-based (red), risk-based (black), outcome-based (blue), and random (green) vaccination strategies.

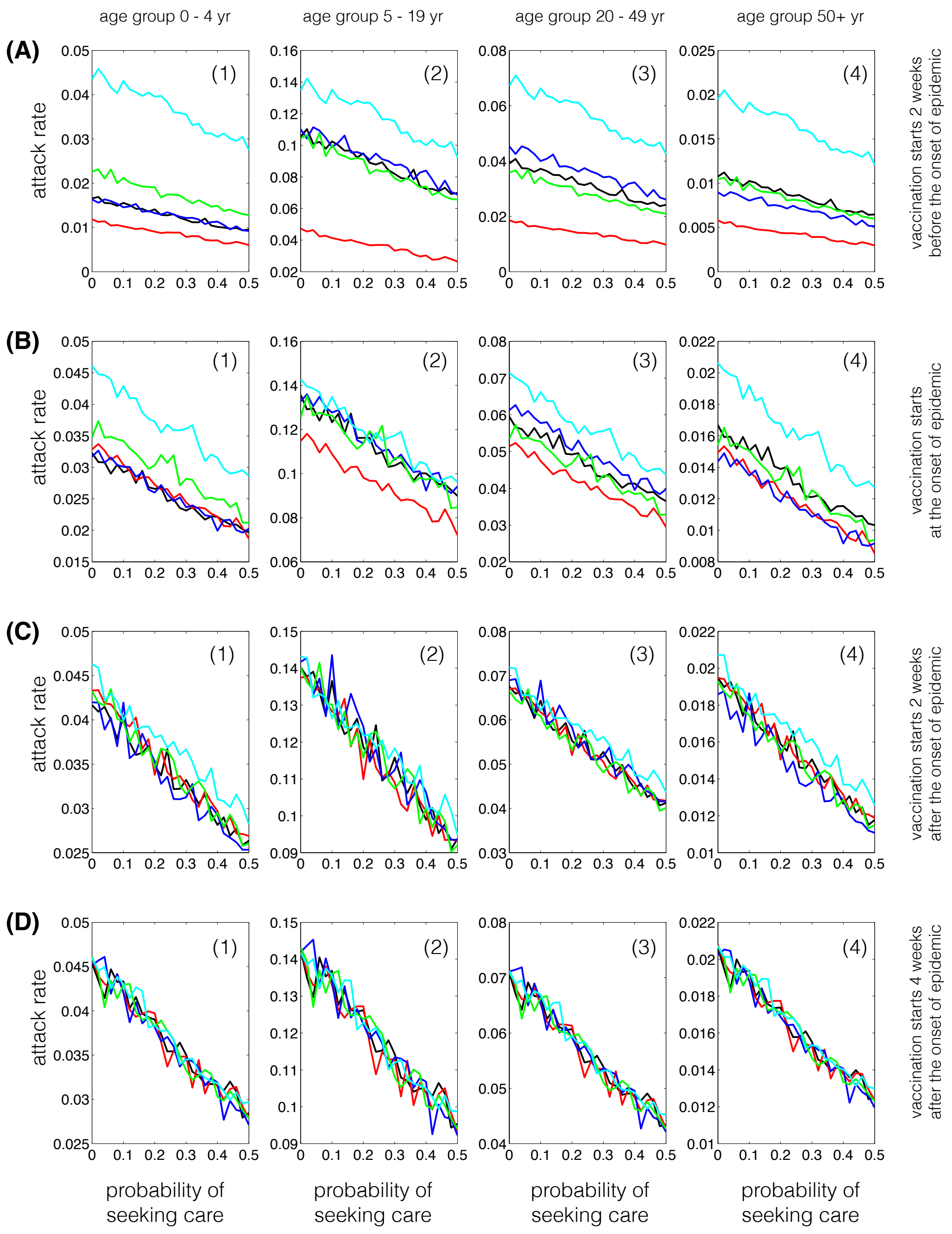

Figure S33. Age-specific attack rates for two-dose vaccination strategies in shifted demographics (remote community). Curves correspond to the scenarios without vaccination (cyan), morbidity-based vaccination strategy (red), risk-based vaccination strategy (black), outcome-based vaccination strategy (blue), and random vaccination strategy (green).

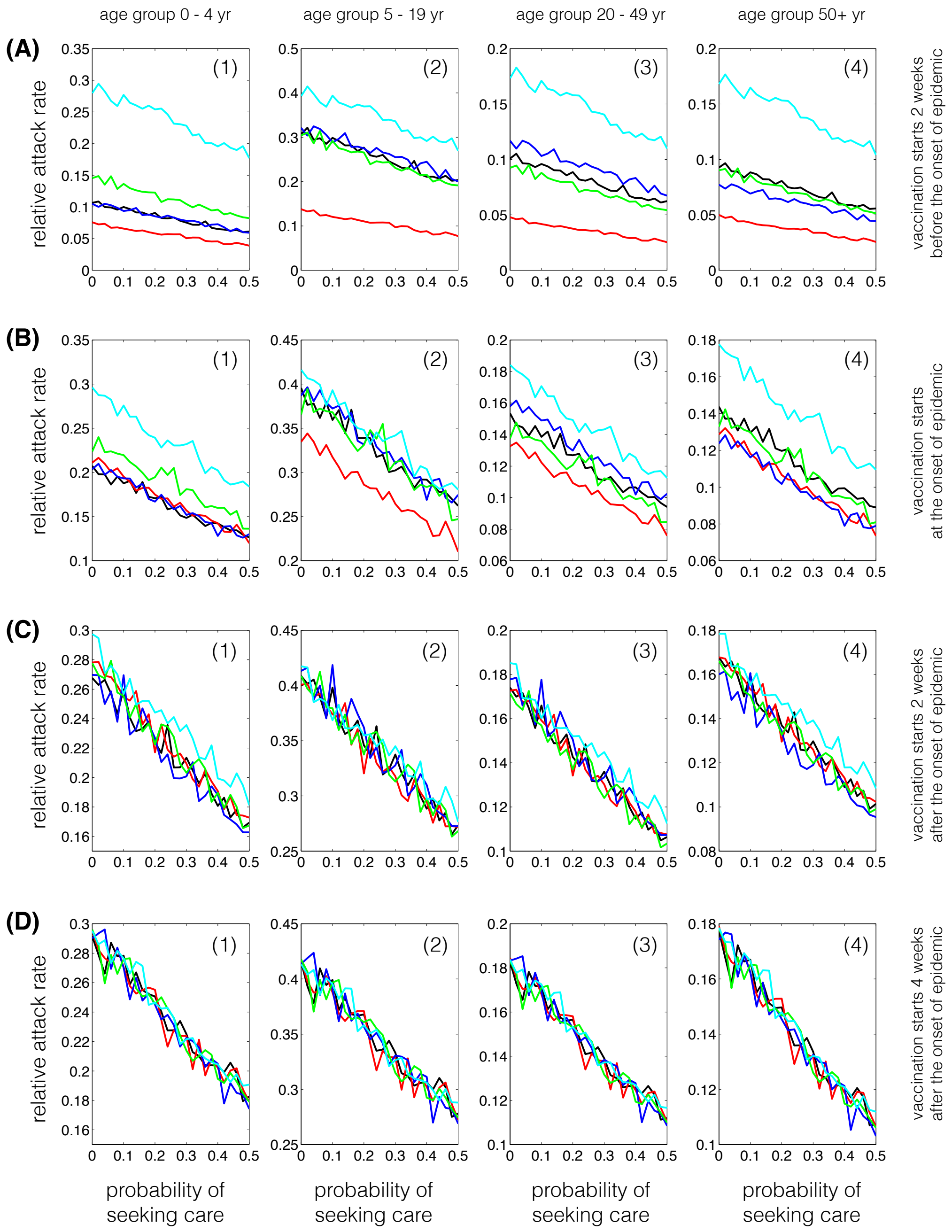

Figure S34. Relative attack rates for two-dose vaccination strategies in shifted demographics (remote community). Curves correspond to the scenarios without vaccination (cyan), morbidity-based vaccination strategy (red), risk-based vaccination strategy (black), outcome-based vaccination strategy (blue), and random vaccination strategy (green).

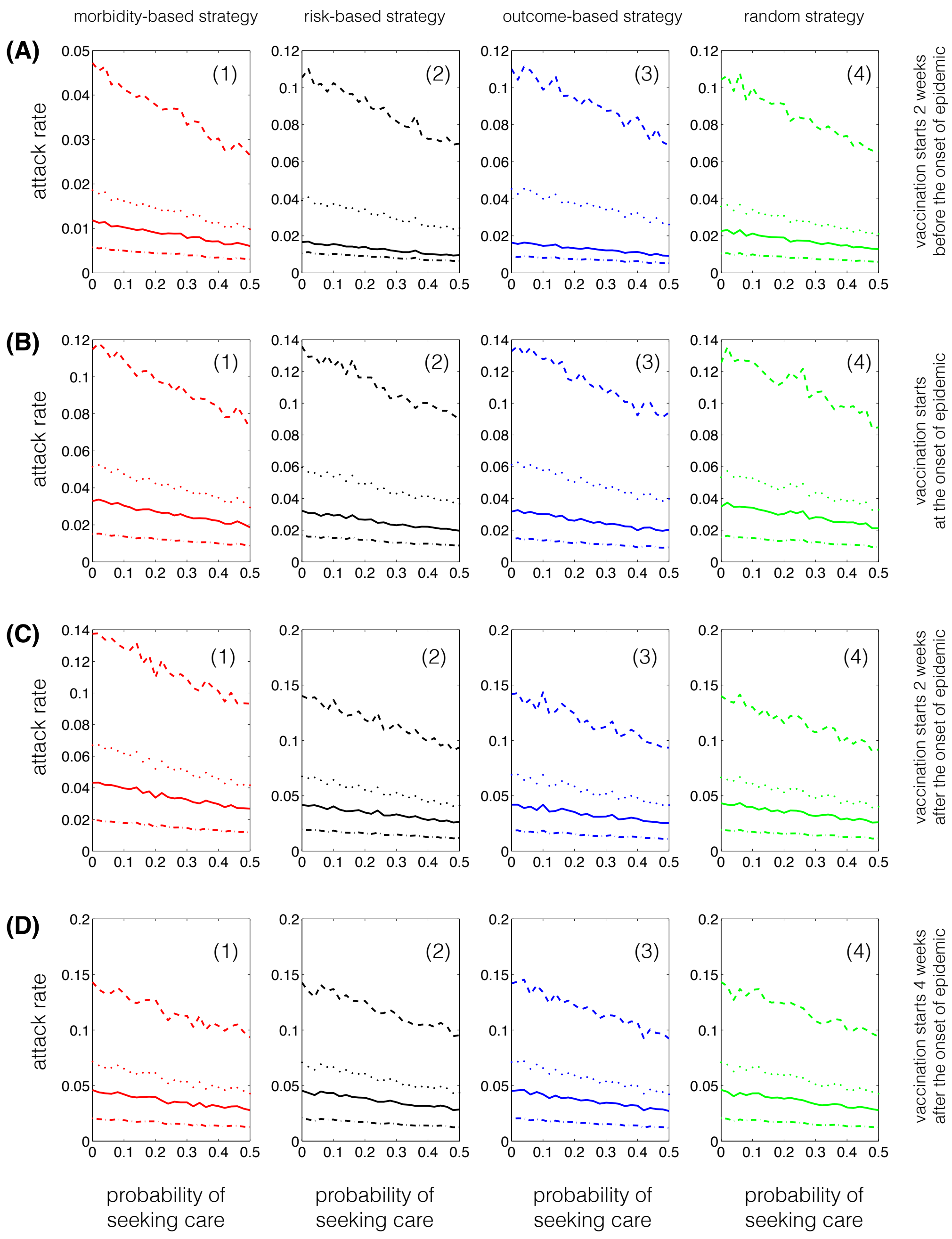

Figure S35. Age-specific attack rates for two-dose vaccination strategies in shifted demographics (remote community) for age groups 0 – 4 (solid curves); 5 – 19 (dashed curves); 20 – 49 (dotted curves); and 50+ (dot-dashed curves). Colours correspond to morbidity-based (red), risk-based (black), outcome-based (blue), and random (green) vaccination strategies.

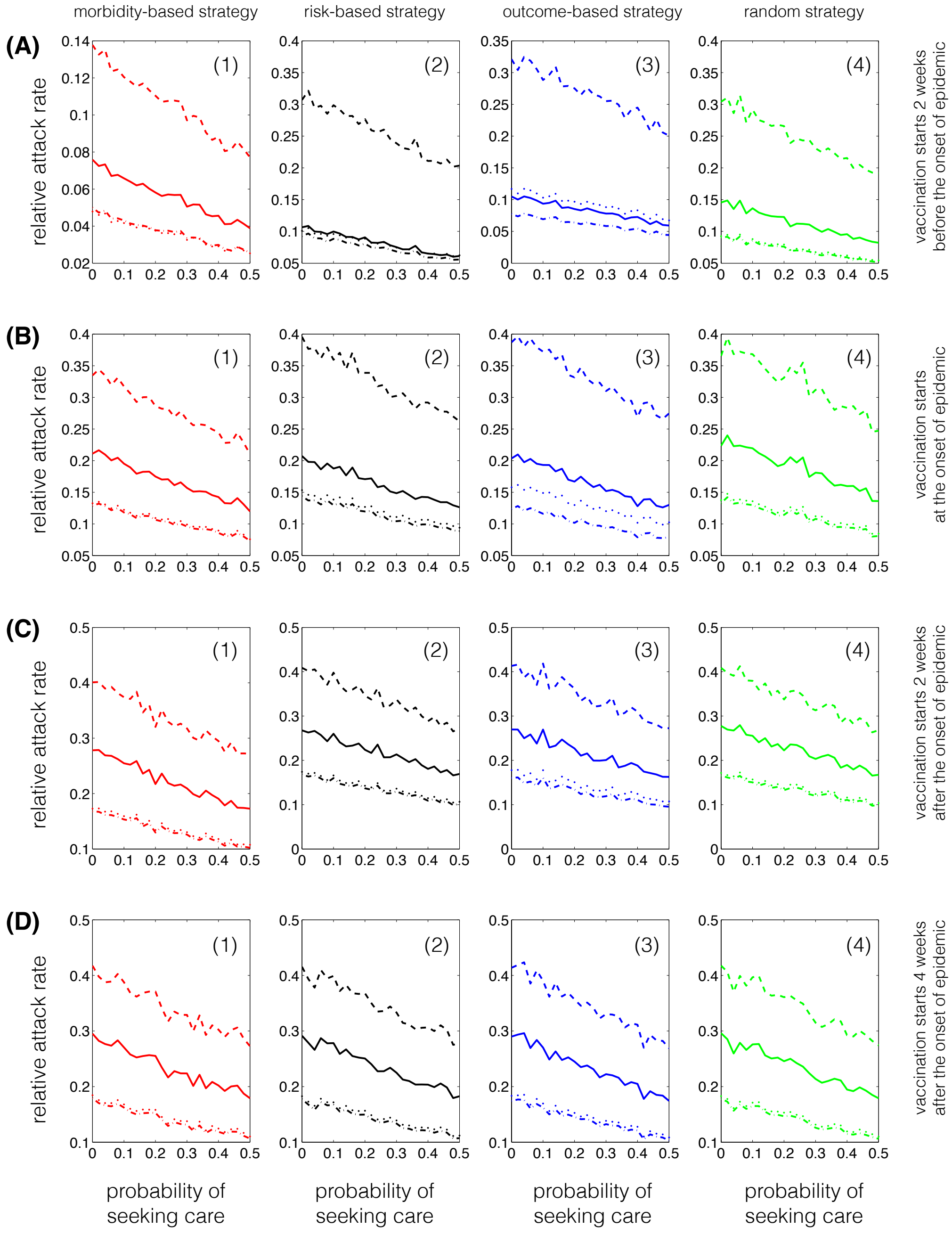

Figure S36. Relative attack rates for two-dose vaccination strategies in shifted demographics (remote community) for age groups 0 – 4 (solid curves); 5 – 19 (dashed curves); 20 – 49 (dotted curves); and 50+ (dot-dashed curves). Colours correspond to morbidity-based (red), risk-based (black), outcome-based (blue), and random (green) vaccination strategies.

13. Hospitalization and treatment ratios

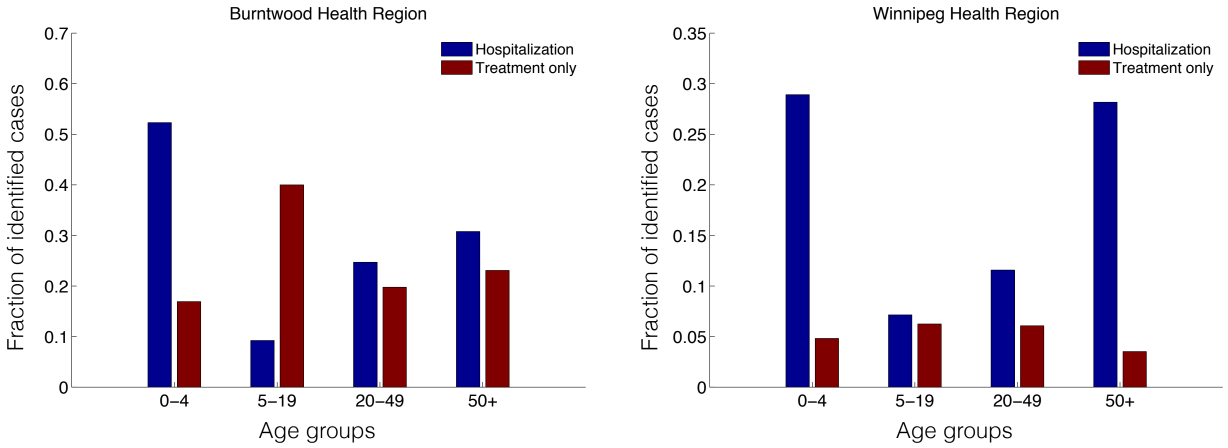

Figure S37. Fraction of identified (laboratory confirmed) cases, either hospitalized or treated (without hospitalization) in the Burntwood and Winnipeg health regions (Manitoba) during the first wave of the 2009 H1N1 pandemic. In each age group, a fraction of confirmed cases were neither hospitalized nor treated.

14. Model parameters

Table S1. Description of model parameters and their values (ranges) used for simulations.

Parameter Description	Value, Range	Notes	Reference, Assumption
Infection transmission probability (per hour)	Calibrated for a given R_0	Baseline probability of infection transmission between one fully susceptible and a symptomatically infectious individual (without treatment) if they are in contact for one hour	(28,22-24)
Mean infectious period (days)	3.38	Infectious period is sampled from a log-normal distribution	(24,41)
Mean latent period (days)	1.5	Latent period is sampled from a uniform distribution	(33,40)
Mean pre-symptomatic period (days)	0.5	Pre-symptomatic period is sampled from a log-normal	(33,41)

		distribution	
Delay in seeking care (days)	0.5 – 3.5	This delay is sampled from a uniform distribution in this range	(32,41)
Infectiousness of pre-symptomatic stage relative to symptomatic stage	0.5	Pre-symptomatic is 50% less infectious than symptomatic	(41)
Probability of symptomatic infection seeking treatment	0 – 0.5	The probability that a symptomatic individual will seek treatment	Varied in scenarios
Probability that an agent younger than 19 years of age practices self-isolation when develops symptomatic infection	0.8	School and pre-school age groups	(31,33)
Probability that an agent aged 19 – 49 practices self-isolation when develops symptomatic infection	0.3	Adults	(26,28)
Probability that an agent older than 50 years of age practices self-isolation when develops symptomatic infection	0.8	Older adults	(26,28)
Treatment duration (days)	The same as infectious period as sampled from the log-normal distribution	Entire infectious period for each individual	Assumed
Reduction of disease transmissibility for infectious agents receiving treatment compared to untreated cases	0.6	Reduction in probability of transmitting the disease since start of the treatment	(28,33-35,41,42,43)
Vaccine clinic hours	09:00 – 17:00	Vaccines are administered during these hours	Assumed

Vaccine doses available per day	1.7% of the total population	Distributed according to the vaccine strategy	Assumed
Probability of developing asymptomatic infection in the absence of vaccine	0.4	Affects probability of developing asymptomatic infection	(28,33,35,41,42,43)
High efficacy vaccine		Age-specific vaccine effectiveness	(15-18,34,44)
Age groups			
0 – 4 years	0.6 – 0.9		
5 – 19 years	0.6 – 0.9		
20 – 49 years	0.6 – 0.9		
50+ years	0.4 – 0.7		
Low efficacy vaccine		Age-specific vaccine effectiveness	Assumed, (26)
Age groups			
0 – 4 years	0.1 – 0.3		
5 – 19 years	0.1 – 0.3		
20 – 49 years	0.1 – 0.3		
50+ years	0.1 – 0.3		
Probability of hospitalization for shifted demographics (remote community)		Probability of hospitalization due to symptomatic infection	Obtained from epidemiological data of H1N1pdm09
Age groups			
0 – 4 years	0.52		
5 – 19 years	0.09		
20 – 49 years	0.24		
50+ years	0.31		
Probability of hospitalization for urban centre		Probability of hospitalization due to symptomatic infection	Obtained from epidemiological data of H1N1pdm09
Age groups			
0 – 4 years	0.29		
5 – 19 years	0.07		
20 – 49 years	0.11		
50+ years	0.28		
Vaccine-induced protection timelines	2 weeks	Time it takes for a vaccine to induce the protection level sampled from the	(26,27,45)

associated range

15. Disease model states

Table S2. Disease states in the model.

State variable	Current state	Moving to Next State
S	Susceptible	$\boldsymbol{E}$ if exposed, or $\boldsymbol{S_V}$ if vaccinated
S_V	Susceptible and vaccinated	$\boldsymbol{E_W}$ if exposed before the vaccine boosting period (72 hours) elapses, and $\boldsymbol{E_V}$ otherwise
E	Exposed to infection	$\boldsymbol{P}$ when latent period ends or $\boldsymbol{E_W}$ if vaccinated
E_V	Exposed to infection, vaccinated	$\boldsymbol{P_V}$ when latent period ends
E_W	Exposed to infection, vaccine wastage	$\boldsymbol{P_W}$ when latent period ends
P	Pre-symptomatic infection	$\boldsymbol{I_{US}}$, $\boldsymbol{I_{UK}}$, or $\boldsymbol{A}$ when pre-symptomatic period ends, $\boldsymbol{P_W}$ if vaccinated
P_V	Pre-symptomatic infection, vaccinated	$\boldsymbol{I_{US}}$, $\boldsymbol{I_{UK}}$, or $\boldsymbol{A_V}$ when pre-symptomatic period ends
P_W	Pre-symptomatic infection, vaccine wastage	$\boldsymbol{I_{USW}}$, $\boldsymbol{I_{UKW}}$, or $\boldsymbol{A_W}$ when pre-symptomatic period ends
I_{UK}	Infectious, untreated, keeping regular schedule	$\boldsymbol{I_{TK}}$ if seeks care and receives treatment, $\boldsymbol{R_U}$ when infectious period ends, $\boldsymbol{I_{UKW}}$ if vaccinated, or $\boldsymbol{H}$ if hospitalized
I_{UKV}	Infectious, untreated, keeping regular schedule, vaccinated	$\boldsymbol{I_{TKV}}$ if seeks care and receives treatment, $\boldsymbol{R_{UV}}$ when infectious period ends, or $\boldsymbol{H_V}$ if hospitalized
I_{UKW}	Infectious, untreated, keeping regular schedule, wasted vaccine	$\boldsymbol{I_{TK}}$ if seeks care and receives treatment, $\boldsymbol{R_{UW}}$ when infectious period ends, or $\boldsymbol{H_W}$ if hospitalized

I_{US}	Infectious, untreated, self-isolated	$\boldsymbol{I_{TS}}$ if seeks care and receives treatment, $\boldsymbol{R_U}$ when infectious period ends, $\boldsymbol{I_{USW}}$ if vaccinated, $\boldsymbol{H}$ if hospitalized
I_{USW}	Infectious, untreated, self-isolated	$\boldsymbol{R_W}$ if infectious period ends, $\boldsymbol{H_W}$ if hospitalized, $\boldsymbol{I_{TSW}}$ if receives treatment
I_{USV}	Infectious, untreated, self-isolated, vaccinated	$\boldsymbol{I_{TSV}}$ if seeks care and receives treatment, $\boldsymbol{R_V}$ if infectious period ends, $\boldsymbol{H_V}$ if hospitalized
I_{TKV}	Infectious, treated, keeping regular schedule, vaccinated	$\boldsymbol{R_{TV}}$ when infectious period ends, $\boldsymbol{H_V}$ if hospitalized
I_{TKW}	Infectious, treated, keeping regular schedule, vaccine wasted	$\boldsymbol{R_{TW}}$ when infectious period ends, $\boldsymbol{H_W}$ if hospitalized
I_{TS}	Infectious, treated, self-isolated	$\boldsymbol{R_T}$ when infectious period ends
I_{TSW}	Infectious, treated, self-isolated, vaccine wasted	$\boldsymbol{R_{TW}}$ when infectious period ends, $\boldsymbol{H_W}$ if hospitalized
I_{TSV}	Infectious, treated, self-isolated, vaccinated	$\boldsymbol{R_{TV}}$ when infectious period ends, $\boldsymbol{H_V}$ if hospitalized
A	Asymptomatic	$\boldsymbol{R_U}$ or $\boldsymbol{A_W}$ if vaccinated
A_W	Asymptomatic, wasted vaccine	$\boldsymbol{R_{WU}}$
A_V	Asymptomatic, vaccinated	$\boldsymbol{R_{VU}}$
H	Hospitalized	$\boldsymbol{R_T}$ when infectious period ends
H_V	Hospitalized, vaccinated	$\boldsymbol{R_{TV}}$ when infectious period ends
H_W	Hospitalized, wasted vaccine	$\boldsymbol{R_{TW}}$ when infectious period ends

R_T	Recovered, treated (identified)	None
R_U	Recovered, untreated	$\boldsymbol{R_{UW}}$ if receives vaccine
R_{TW}	Recovered, treated, wasted vaccine	None
R_{TV}	Recovered, treated, vaccinated	None
R_{UW}	Recovered, untreated, wasted vaccine	None
R_{UV}	Recovered, untreated, vaccinated	None
